# Genome-Wide Association Studies of 39 Seed Yield-Related Traits in Sesame (*Sesamum indicum* L.)

**DOI:** 10.3390/ijms19092794

**Published:** 2018-09-17

**Authors:** Rong Zhou, Komivi Dossa, Donghua Li, Jingyin Yu, Jun You, Xin Wei, Xiurong Zhang

**Affiliations:** 1 Key Laboratory of Biology and Genetic Improvement of Oil Crops, Oil Crops Research Institute of the Chinese Academy of Agricultural Sciences, Ministry of Agriculture, No. 2 Xudong 2nd Road, Wuhan 430062, China; rongzzzzzz@126.com (R.Z.); dossakomivi@gmail.com (K.D.); ldh360681@163.com (D.L.); yujingyin@caas.cn (J.Y.); youjunbio@163.com (J.Y.); 2 Centre d’Etude Régional Pour l’Amélioration de l’Adaptation à la Sécheresse (CERAAS), Route de Khombole, Thiès, Thiès Escale Thiès BP3320, Senegal; 3 College of Life and Environmental Sciences, Shanghai Normal University, Shanghai 200234, China

**Keywords:** sesame, genome-wide association study, yield, QTL, candidate gene

## Abstract

Sesame is poised to become a major oilseed crop owing to its high oil quality and adaptation to various ecological areas. However, the seed yield of sesame is very low and the underlying genetic basis is still elusive. Here, we performed genome-wide association studies of 39 seed yield-related traits categorized into five major trait groups, in three different environments, using 705 diverse lines. Extensive variation was observed for the traits with capsule size, capsule number and seed size-related traits, found to be highly correlated with seed yield indexes. In total, 646 loci were significantly associated with the 39 traits (*p* < 10^−7^) and resolved to 547 quantitative trait loci QTLs. We identified six multi-environment QTLs and 76 pleiotropic QTLs associated with two to five different traits. By analyzing the candidate genes for the assayed traits, we retrieved 48 potential genes containing significant functional loci. Several homologs of these candidate genes in *Arabidopsis* are described to be involved in seed or biomass formation. However, we also identified novel candidate genes, such as *SiLPT3* and *SiACS8*, which may control capsule length and capsule number traits. Altogether, we provided the highly-anticipated basis for research on genetics and functional genomics towards seed yield improvement in sesame.

## 1. Introduction

The use of high-quality oil in human daily food intake is an important part of overall well-being. Sesame (*Sesamum indicum* L.) is a source of an excellent vegetable oil rich in vital minerals, vitamins, phytosterols, polyunsaturated fatty acids, tocopherols and unique classes of lignans such as sesamin and sesamolin, which have been identified as beneficial compounds for human health [1]. Moreover, its seeds have one of the highest oil contents (55%) among major oilseed crops, as well as a high protein content [2]. The world population is growing fast and the demand for vegetable oil in quantity and high-quality is pressing. Vegetable oil consumption is expected to double by 2040 [3]. Therefore, sesame can play a significant role in satisfying this demand.

Sesame is essentially a small-scale farmer crop and its cultivation offers two main advantages: it is a very rewarding crop because of its low production cost and high sale price; and, it is also a very resilient crop, able to provide yield and generate incomes in marginal areas where many other crops cannot grow [4,5]. Over the last decade, the production of sesame seeds has doubled and the growing area has extended to more than 50 countries in the world, showing an ever-increasing interest in this crop [6]. However, sesame has a very low seed yield capacity compared to other oilseed crops [7]. According to the Food and Agriculture Organization, the average seed yield of sesame was only 578 kg/ha in 2016, ranked as the second lowest among the major oil crops [6]. Therefore, understanding the genetic basis of seed yield-related traits and applying that knowledge in sesame breeding programs might be instrumental in developing stable high-yielding sesame varieties.

The yield of any crop is a complex character, which depends upon many independent contributing components. Deep understanding of the relationship between yield and its components is crucial to the selection process and to crop improvement [8]. Sesame seed yield per plant is considered to mainly have three components, namely, the number of capsules per plant, the number of seeds per capsule and seed weight. Some other factors, including plant height, capsule dimensions, the first capsule axis height and the number of internodes, were found to be strongly associated with seed yield in sesame [9,10]. In addition, the plant growth habit, branching type, capsule shattering, management practices, and biotic and environmental factors can significantly affect sesame yield [11]. Beside the variation among cultivars for seed yield components, the within-plant variation is extremely important. For example, some sesame cultivars can have three or more capsules per leaf axil. Mosjidis and Yermanos [12] observed that seed weight from medial capsules is higher than that from lateral capsules. Moreover, Tashiro et al. [13] and later Kumazaki et al. [14] confirmed the significant differences between seed weight between capsules from nodes located at different positions along the main stem within the same plant. Accordingly, dissecting the genetic basis of the seed yield components in sesame may be challenging and will need meticulous analysis of the multiple and complex seed yield components.

Thirteen quantitative trait loci (QTL) were detected for seven seed yield-related traits using the linkage mapping approach in sesame [10]. Genome-wide association study (GWAS) has proven to be advantageous over bi-parental QTL mapping as it captures greater diversity and offers higher resolution for gene and favorable allele discovery in several plant species [15]. Recently, GWAS was also successfully applied to sesame to unravel the genetic basis of the oil production and quality traits, yield related traits, important agronomic traits, as well as salt and drought tolerance [16,17]. The objective of the hereby study was to employ the GWAS approach to comprehensively decipher the genetic basis of 39 seed yield-related traits in sesame and unlock potential alleles and genes for seed yield improvement based on a large and diverse sample phenotyped in three different environments.

## 2. Results

### 2.1. Variability and Correlation of the Seed Yield-Related Traits in the Sesame Association Panel

**Figure 1:**
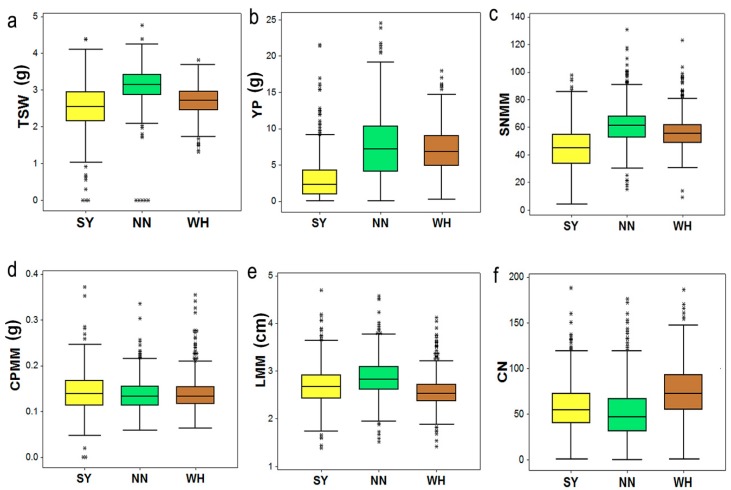
Boxplots displaying variation of six traits across three different environments (SY = Sanya, NN = Nanning and WH = Wuhan). Definition of the labels can be found at the end of this article.

A total of 39 direct and indirect seed yield-related traits were studied and classified into five main trait groups: yield index, seed traits, capsule number, capsule size, and capsule pericarp (Table S1). Ten yield-related traits that were investigated in the previous research of Wei et al. [16] were also included in this study. Descriptive statistics for the traits across the 705 accessions included in this study are listed in the Table S2. Overall, the sesame diversity panel exhibited extensive trait variation across the three environments analyzed (Figure 1 and Figure S1). We selected three contrasting environments for phenotyping (Nanning (NN), Wuhan (WH) and Sanya (SY)) because they represent natural sesame growing areas in China and also cover different geographical regions of China: Central China (WH), South China (SY), Southwest China (NN). The traits appeared to be slightly higher at NN environment compared with WH and SY, but overall the yields are similar among the three locations. Some traits, especially those related to the capsule number and capsule size groups, were stable across environments; however, the traits belonging to the yield index group displayed a high variation. This observation was further confirmed with the broad-sense heritability estimates (Table S2). Generally, a large portion of the phenotypic variance in seed yield components could be attributed to the genotypic effects in sesame.

**Figure 2:**
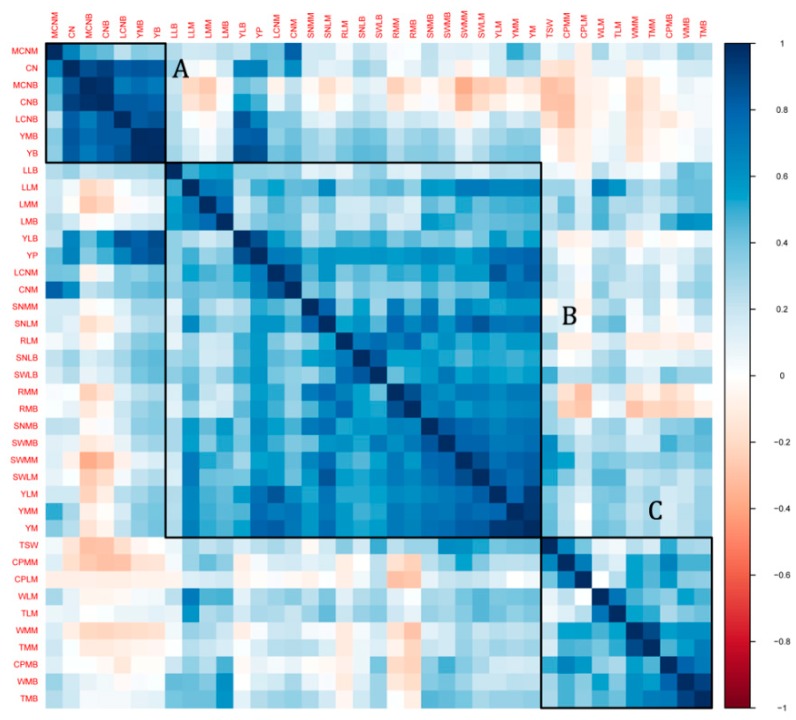
Correlation between all the seed yield-related traits in sesame. Blue color depicts positive correlation while red color means negative correlation. A, B and C correspond to the clusters of traits. Definition of the labels can be found at the end of this article.

To gain insight into the relationship between the seed yield-related traits, a clustering and correlation analysis was performed (Figure 2). It can be obviously observed that traits from the same group clustered closely, indicating strong correlations with each other. Furthermore, clustering analysis of the phenotype data highlighted three main groups (A, B and C). Group A comprised capsule number (MCNM, CN, MCNB, CNB and LCNB) and yield index (YMB and YB) related traits, which were strongly and positively correlated. This result shows that a high capsule number in a sesame plant leads to a high yield. The second group (B) was composed of mixed traits in relation to yield index, seed traits, and capsule size. From such a cluster, we inferred that accessions with high ratios of seed weight/capsule weight are likely to have a high yield. In addition, we found that high values of seed number and seed weight-traits are favorable for seed yield in sesame. Finally, Group C clustered some capsule pericarp and capsule size-related traits with moderate correlation values. Since no yield index trait was observed in this group, we concluded that it may not directly contribute to seed yield in sesame. More importantly, we found that traits from this group were negatively correlated with traits contributing to a high seed yield in sesame. For example, accessions with high capsule pericarp thickness have lower yield indexes.

### 2.2. Genetic Variants Associated with Seed Yield-Related Traits in Sesame

**Figure 3:**
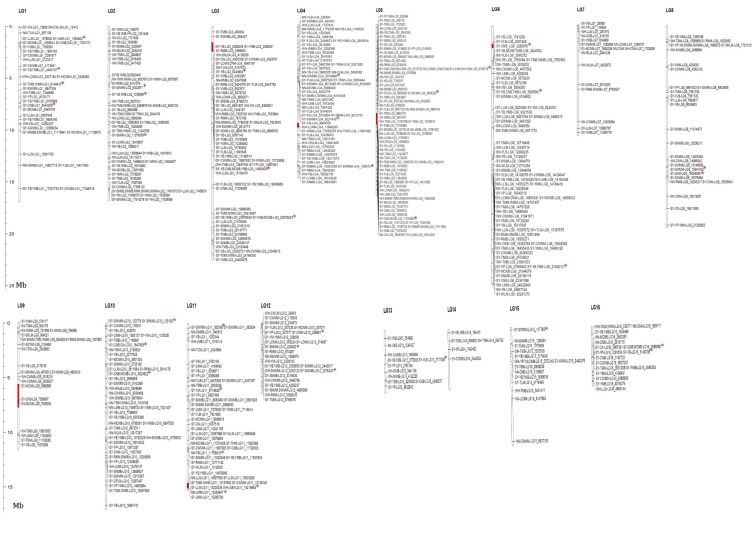
Genomic location of the 547 QTLs identified for seed yield-related traits in sesame. QTLs were named as follow: ENVIRONMENT-TRAIT-LINKAGEGROUP_POSITION. Bars represent the linkage groups of sesame genome. Red portions of the bars represent the previous QTLs detected by Wu et al. [10]. Red stars represent loci previously detected by Wei et al. [16]. Definition of the labels can be found at the end of this article.

**Figure 4:**
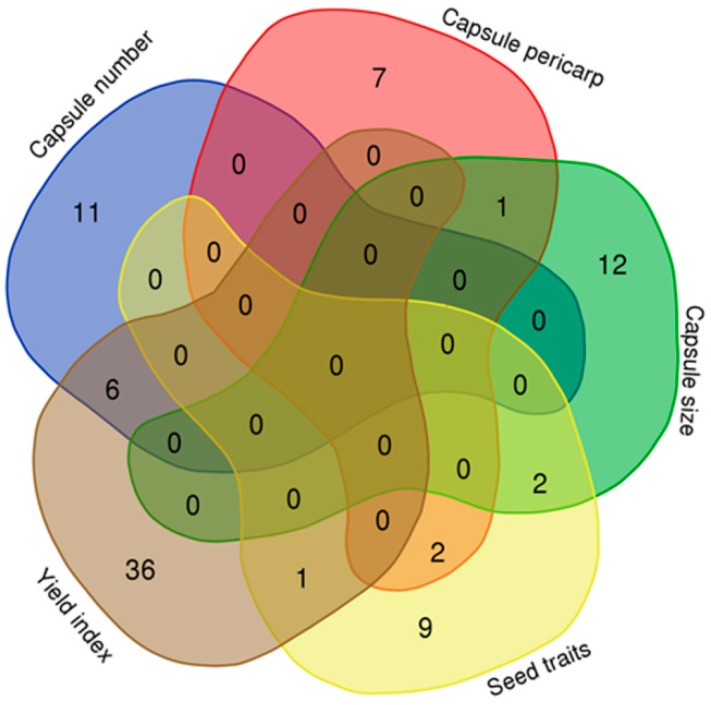
Venn diagram depicting the shared and common QTLs between five groups of seed yield-related traits analyzed in this study.

**Figure 5:**
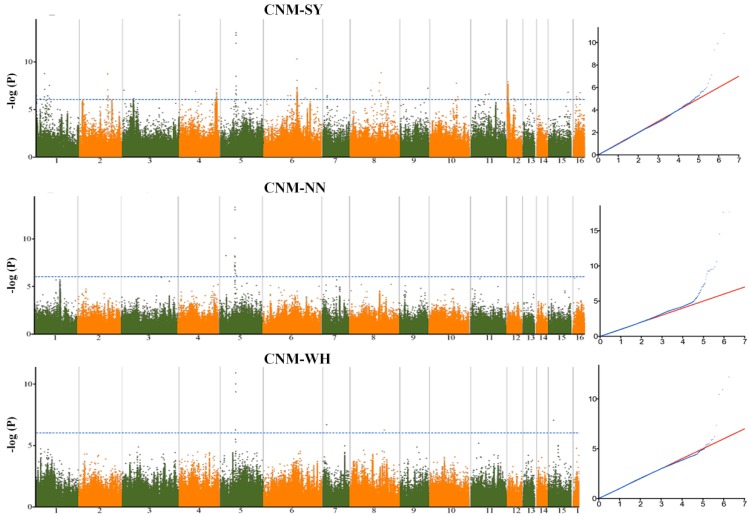
Genome-wide association mapping of effective capsule number in main stem (CNM) in sesame from three different environments (SY = Sanya, NN = Nanning and WH = Wuhan).

**Figure 6:**
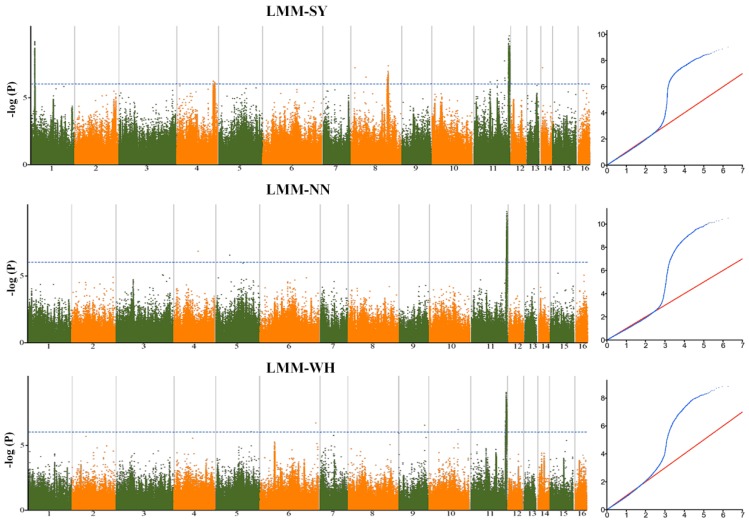
Genome-wide association mapping of length of medial capsule in main stem (LMM) in sesame from three different environments (SY = Sanya, NN = Nanning and WH = Wuhan).

To predict significant marker-trait associations for seed yield-related traits, the mixed model was implemented in this study of the phenotype data from each environment. Genome wide association studies (GWAS) revealed 646 statistically significant loci (*p* < 10^−7^) across the three environments associated with the 39 traits. A total of 6% of the loci were in line with the previous identified yield-related loci [16]. Significant loci were found on all of the 16 linkage groups (LG) of the genome, justifying the complex genetic architecture of the seed yield in sesame. The highest number of significant loci (86) was detected on the LG5, while the LG14 harbored only six significant loci (Table S3, Figure S2). The phenotypic variation explained by the lead loci ranged from 6.01 (SNP2372143) to 17.9% (SNP6737753 and SNP5479753), suggesting a moderate contribution to the traits (Table 1). We defined as a QTL the 88 kb region (corresponding to the linkage disequilibrium (LD) window) surrounding the peak loci and containing at least three significant loci [17]. By combining peak single nucleotide polymorphism (SNP)-trait-environment, a total of 547 QTLs were identified (Figure 3). Furthermore, by comparing peak loci through environments and traits, we uncovered six stable QTLs (detected in different environments for the same trait) and 76 pleiotropic QTLs associated with two to five various traits (Table 1). We compared the detected pleiotropic QTLs between the five groups of traits defined in this study. The results showed that most of the pleiotropic QTLs principally controlled traits from the same group (Figure 4). Few common QTLs could be observed between pairs of trait groups and there was no shared QTL for more than three traits groups. Overall, these results corroborate the phenotypic relationships observed in Figure 2. For example, there is no common QTL for the capsule pericarp and yield index groups; similarly for the capsule size and yield index groups. Conversely, the trait groups related to the yield index and capsule number exhibited the highest number of common QTLs (6), demonstrating that these groups shared similar genetic architectures. The examples presented in Figure 5 and Figure 6, related to the trait-association for the effective capsule number in the main stem (CNM) and length of medial capsule in the main stem (LMM) of the three environments, highlight two stable QTLs detected on LG5 for CNM and LG11 for LMM. Overall, more significant loci were discovered in SY compared to the other environments.

### 2.3. Comparing Previous QTLs on Seed Yield-Related Traits from Bi-Parental Linkage Mapping with Our GWAS Results

In a previous study, Wu et al. [10] constructed a high-density genetic map of sesame using a population of 224 recombinant inbred lines based on the restriction-site associated DNA sequencing (RAD-seq) approach and identified several seed yield-related QTLs (plant height, first capsule height, capsule axis length, capsule number per plant, capsule length, seed number per capsule and thousand seed weight). Four similar traits, viz., capsule number per plant, capsule length, seed number per capsule and thousand seed weight, were also investigated in our study and we compared both studies to identify common genomic regions. The physical locations of the QTLs were searched on the reference genome [18] following the descriptions of Dossa [19]. Six QTLs detected by Wu et al. [10] matched with regions around significant loci detected in this study (Table 2; Figure 3). Interestingly, we observed a good consistency between the traits related to those six QTLs and the traits associated with the corresponding significant loci. For example, the capsule length QTL (Qcl-12) from Wu et al. [10] corresponded to nine loci associated with capsule size-related traits in our study. Also, the QTL Qcn-11 for capsule number per plant covered three significant loci identified for capsule number based on our GWAS. Another important finding is that the overlapped QTLs from Wu et al. [10] can be pleiotropic since they expanded on several significant loci which were associated with various seed yield traits in our study.

### 2.4. Important Candidate Genes Associated with Seed Yield in Sesame

**Figure 7:**
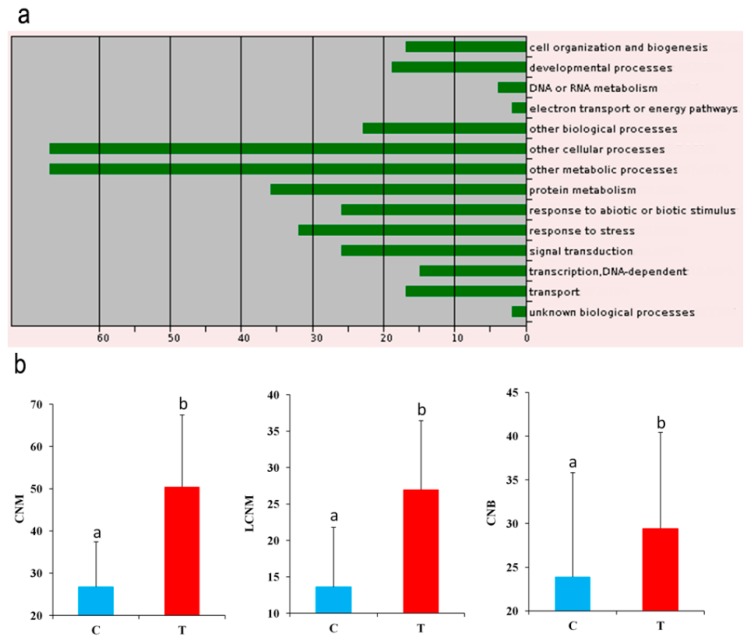
Functional analysis of 48 candidate gene-containing significant SNPs. (**a**) Biological function of the SNP-containing genes. (**b**) Identification of the favorable allele for the gene *SiACS8*. 262 genotypes harboring the C allele and 420 harboring the T allele were used. Different letters above bars represent significant difference (*p* < 0.05) between genotypes. The error bar indicates the standard error of the mean. Definition of the labels can be found at the end of this article.

To identify the candidate genes controlling the seed yield-related traits in sesame, all the genes in 88 kb around the peak loci were retrieved [17]. In total, 7149 genes were identified and the number of genes in the LD window ranged between 8 and 42 (Table S4). Within these genes, 48 contained significant loci (Table S5). We particularly focused on these SNP-containing genes as they are more likely to modulate seed yield in sesame. Their homologs in *Arabidopsis* were identified and their functions predicted. Gene ontology analysis of these genes indicated that they are involved in developmental process, DNA and protein metabolism, response to stress, signal transduction, cell organization and biogenesis, transport and transcription (Figure 7a). Several homolog genes in *Arabidopsis* are well known to be directly or indirectly implicated in seed yield and biomass production. For example, the gene *AGL20* (AGAMOUS-like 20) plays an important role in flowering time [20], hence is directly associated with seed yield in *Arabidopsis*. In this study, we detected an intronic SNP located in the gene *SIN_1013997* (homolog of *AGL20*) strongly associated with the branch per plant seed yield and with the medial capsules in branch seed yield. Another important illustration concerns the gene *SIN_1006338* (*SiACS8*), which is located in the pleiotropic QTL associated with four various traits and was detected in all the three environments. A non-synonymous polymorphism (T/C) at the position 6,738,735 bp in this gene modulates the capsule number related traits (LCNM, CNM and CNB). An in-depth analysis suggests that the thymine allele is the favorable allele as it increases the capsule number on the stem and, therefore, leads to a higher yield (Figure 7b). Furthermore, the frequency of the T allele was rapidly increased by recent breeding, from 57% in landraces to 92% in modern cultivars. The gene *SiACS8* was previously identified as being associated with the capsule number per axil, particularly controlling the 1:3 capsules per axil in sesame [16]. These results further support our findings, indicating that *SiACS8* is indeed the causative gene controlling the capsule number trait in sesame. The homolog of *SiACS8* in *Arabidopsis AT4G37770* (*AtACS8*) was reported to be an auxin-induced gene involved in ethylene biosynthesis, suggesting that the number of capsules on sesame stem is under the regulation of plant hormones [21].

A total of seven genes (*SIN_1017946*, *SIN_1017109*, *SIN_1021838*, *SIN_1019958*, *SIN_1011780*, *SIN_1019747* and *SIN_1014519*) involved in nutrient assimilation, carbohydrate metabolism, repression of early auxin response and kinase activity contain significant loci strongly associated with the total seed yield per plant (YP). These genes appear to be important in an effective source/sink relationship favorable for a high yield in sesame.

**Figure 8:**
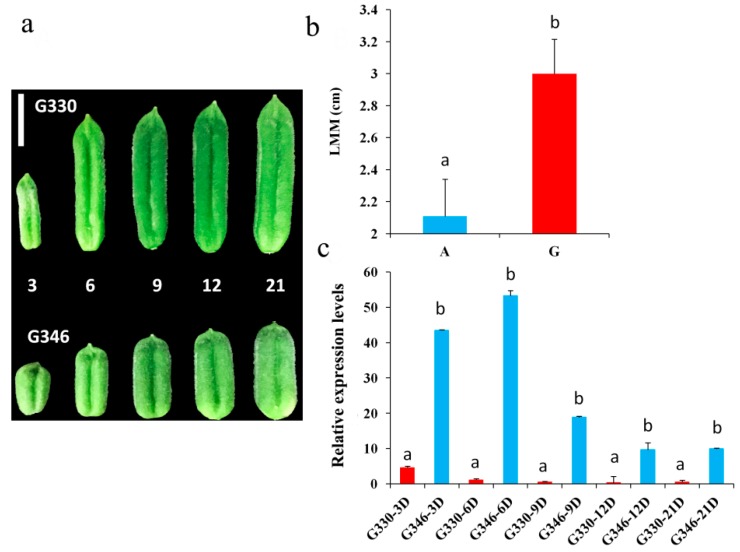
Expression analysis of the candidate gene for LMM trait between two contrasting accessions. (**a**) Phenotypes of G330 and G346 displaying long and short capsule length, respectively, at 3, 6, 9, 12 and 21 days after pollination. (**b**) Identification of the favorable allele at the locus 15,219,964 bp on the LG11. A total of 427 genotypes harboring the A allele and 175 harboring the G allele were used. (**c**) qRT-PCR relative expression level of the gene *SIN_1010995* between G330 and G346 at different days after pollination. Different letters above bars represent significant difference (*p* < 0.05) between genotypes. The error bar indicates the standard error of the mean. The sesame *Actin* gene (*SIN_1006268*) was used as the internal reference and 3 biological replicates and 3 technical replicates were used.

Some strongly associated loci were not located in the genic region; hence, gene expression analysis can give clues to pinpoint the probable candidate genes. As a proof of concept, we focused on the trait LMM and investigated the associated candidate gene. The strongest significant loci (A/G) (−log_10_(*p*) = 9.06) for LMM was located on the LG11 at the position 15,219,964 bp. Accessions with the guanine allele have a long capsule size as opposed to accessions with the adenosine allele. Interestingly, the frequency of the G allele in modern cultivars (20%) is comparable with that of landraces (37%), implying that this allele has not yet been intensively selected. Three genes *SIN_1011000*, *SIN_1010995* and *SIN_1010983* were found in the linkage disequilibrium window. Judging from the quantitative real time PCR (qRT-PCR) expression analysis of these genes, only *SIN_1010995* displayed a conspicuous discrepancy between the short and long capsule size accessions at different developmental stages (Figure 8). The expression level of *SIN_1010995* (*SiLPT3*), a lipid transfer protein, was striking in the short capsule size accession but weakly expressed in the long capsule size accession. LPT3 proteins are described to be involved in cell wall edification, and more precisely in biosynthesis of cutin, which has been proposed to regulate cell adhesion during plant development [22]. The homolog gene of *SiLPT3* in *Arabidopsis AT5G59320.1* (*AtLPT3*) exhibited higher expression in the silique than other organs of *Arabidopsis*, indicating an active role in silique development [23]. Based on these observations, we speculate that *SiLPT3* regulates cell adhesion in the sesame capsule that contributes to the capsule length.

## 3. Discussion

The seed yield improvement of sesame is a prerequisite for the rapid expansion of the crop. Although sesame has being cultivated for a long time (~5000 years), few efforts have been made for its improvement [5]. In fact, the lack of basic information on the genetics of important agronomical traits, especially the traits complexly inherited, are causing hindrance for the breeders to achieve higher yields [24]. In this study, we observed a high variability for the assayed seed yield related traits, suggesting that our association panel harbors a large diversity necessary for genome wide association studies (GWAS). In a previous comprehensive GWAS for seed quality traits, Wei et al. [16], using the same association panel, found a low population structure, a moderate linkage disequilibrium (LD) decay (88 kb) and recommended that a high marker density, as employed in our study, could give ample power for association analyses. Several authors have studied traits that contribute to the seed yield formation in sesame. Distinctly, the capsule number per plant is a primary determinant for high seed yield in sesame [7,9,10,25,26]. In fact, sesame seeds grow in a capsule; therefore, more capsules on the plant are likely to yield more seeds [4]. Moreover, the number of seeds per capsule and the seed weight are also largely reported as important contributors to seed yield [10,27,28]. Our results match well with those of the literature, as we found that capsule size, capsule number and seed size-related traits are strongly correlated with yield indexes.

Our GWAS results revealed several clusters of significant loci, highlighting important genomic regions associated with seed yield-related traits. Interestingly, many pleiotropic QTLs were identified but an in-depth analysis indicates that very few QTLs were associated with traits from the different groups (Table S1). These results suggest that seed yield component traits from the same group have a similar genetic architecture but traits from different groups may be manipulated independently to increase the seed yield in sesame. Boyles et al. [29] also reported similar observation in sorghum with no overlapping loci for grain yield components.

The GWAS approach is recognized as a powerful tool to reconnect traits back to the underlying genetics and offers higher resolution than classical linkage mapping [30]. Previously, only one study was performed on the genetics of the sesame seed yield by employing the linkage mapping approach [10]. Comparing our results with the previous QTLs, we identified several overlapping loci associated with similar traits. Our study substantially narrows down these QTL regions which will facilitate the identification of the causal genes. In addition, several loci previously identified by Wei et al. [16] in different environments were also detected in this study, implying that these trait-associations are highly stable and could be very useful to accelerate sesame seed yield improvement efforts.

Transcriptome sequencing has been widely used to estimate gene expression changes and enables the efficiency and accuracy of candidate gene discovery in GWAS [31]. In this study, several candidate genes were retrieved from the genomic regions significantly associated with the assayed traits. To effectively pinpoint the causal genes for seed yield-related traits, additional RNA-seq data could be exploited as demonstrated in *Brassica napus*, maize, cotton, sorghum, etc. [31,32,33]. Nonetheless, genes containing associated SNPs which were detected in this work represent potential candidates for further functional analysis using the transgenic approach [34] and genome-editing technologies using CRISPR/Cas system. Meanwhile, the peak loci could be transformed into allele-specific markers for applications in breeding programs to design sesame varieties with improved seed yield. In fact, Asian, American and European sesame producing countries present higher yields than in Africa [6]. This can be, inter alia, related to the use of elite cultivars. For example, the modern cultivars in our panel have, on average, 70 capsules on the main stem, which is approximately double of the capsule number in landraces, and thus have a higher yield potential. Since several favorable alleles detected in this study have not yet been intensively selected, our GWAS results will undoubtedly assist in incorporating further useful alleles into the elite sesame germplasm for a seed yield increase in the future.

## 4. Materials and Methods

### 4.1. Plant Materials

In the present study, 705 cultivated sesame (*Sesamum indicum* L.) accessions were obtained from the germplasm preserved at the China National Gene Bank, Oil Crops Research Institute, Chinese Academy of Agricultural Sciences (Table S6). The panel is composed of 405 traditional landraces and 95 modern cultivars from China, as well as 205 accessions collected from 28 other countries [16]. All the accessions have been self-pollinated for four generations in Sanya, Hainan province, China (109.187° E, 18.38° N, altitude 11 m).

### 4.2. Field Growth Conditions

Three field trials were set in three environments in China during the years 2013 to 2014 at normal planting seasons [16]. All the accessions were grown at experiment stations in Wuhan (WH), the Hubei province (30.57° N, 114.30° E), Nanning (NN), the Guangxi province (23.17° N, 107.55° E) and Sanya (SY), the Hainan province, (109.187° E, 18.38° N). We recorded ranges of temperature (32–38/25–27 °C, day/night), relative humidity (45–72%) and rainfall (125–210 mm) during the experiment in Wuhan. In Nanning, we recorded ranges of temperature (31–34/25–26 °C, day/night), relative humidity (42–58%) and rainfall (205–235 mm) during our experiment. In Sanya, ranges of temperature (30–33/24–26 °C, day/night), relative humidity (50–75%) and rainfall (159–219 mm) were recorded during our experiment. These data show that Wuhan was the hottest location with the lowest rainfall among the 3 locations. Sanya and Nanning experimental fields have a sandy loam soil while Wuhan experimental field is characterized by a loam soil. The field trials were conducted using a randomized block design with three replications. Each plot had four rows of 2 m long spaced 0.4 m apart. At the four-leaf stage, seedlings were thinned down and eight evenly distributed plants in each row were retained for further analyses. Five uniform plants for each genotype were randomly selected to collect phenotypic data.

### 4.3. Trait Evaluation

Plants at the two ends of each row were not selected to avoid edge effects. Traits evaluated included (1) weight (g), length (cm), width (cm) and thickness (cm) of the dry capsule pericarp and the seed selected from different parts of the plant: medial or lateral position on the main stem or branch; (2) the seed number was counted in capsules from different parts of the plant: medial or lateral position on the main stem or branch; (3) the seed yield (g) was recorded from different parts of the plant: the capsules at medial or lateral position on the main stem or branch, total yields of the main stem, the branch and the whole plant. Based on the seed and capsule pericarp dry weights recorded from different parts of the plant, the ratio seed weight and pericarp weight were also computed. In total, 39 traits were investigated in this study and categorized into five major trait groups: yield index, seed traits, capsule number, capsule size and capsule pericarp (Table S1).

### 4.4. Statistical Analysis

All the statistical analyses were performed using R2.3.0 [35]. For each trait, the least square mean and descriptive statistics such as the minimum, maximum, skewness and kurtosis were estimated based on five replicates in each environment. Variation of the different traits in the different environments was represented as boxplot employing the “ggplot2” package [36]. The broad-sense heritability (*H*^2^) was calculated as follow: *H*^2^ = σ^2^_a_/(σ^2^_a_ + σ^2^_ae_/E + σ^2^_ε_/ER), where σ^2^_a_, σ^2^_ay_, and σ^2^_ε_ are estimates of the variances of accession, accession × environment interaction, and error, respectively, estimated by analysis of variance (ANOVA). E represents Environment, and R is the number of replications. Correlation among the seed yield related traits was estimated by Pearson’s method at a significance level of *p* < 0.05 using the “corrplot” package [37]. For the correlation analysis, we used the best linear unbiased estimator (BLUE) values of phenotype data from the three environments.

### 4.5. Genome Wide Association Study Implementation

The association panel used in the present study was previously fully re-sequenced [16]. A total of 1.8 M common single nucleotide polymorphisms (SNPs) covering the whole genome with minor allele frequency >0.03 were retained for the genome wide association studies (GWAS). Phenotype-genotype association was implemented with the EMMAX model [38]. The matrix of pair-wise genetic distance derived from simple matching coefficients was used as the variance–covariance matrix of the random effect. Using the Genetic type 1 Error Calculator, version 0.2 [39], the effective number of independent SNPs were estimated to be 469,175 and the threshold to declare significant associated loci was approximately *p =* 10^−7^ [16]. Significant associations were also selected on the threshold of *p* ≤ 0.01, corrected for multiple comparisons according to the false discovery rate procedure reported by Benjamini and Hochberg [40].

### 4.6. Candidate Gene Mining

Based on the reference genome [18], all the genes in the 88 kb region corresponding to the average linkage disequilibrium window [16] around the peak associated loci were retrieved. Their homologs in *Arabidopsis thaliana* were predicted and their functions annotated from the database Sinbase 2.0 [18] with a cut off *E*-value of ≤1 × 10^−40^. All the genes containing significant associated loci were prioritized. Moreover, for genomic regions where we did not find any associated SNP-containing genes, the putative candidate genes were retained if the homolog genes in *Arabidopsis thaliana* were described to be involved in seed yield or biomass formation. Gene ontology analysis of the candidate genes was performed using the Blast2GO tool v.3.1.3 [41] and plotted with the WEGO tool [42].

### 4.7. Gene Expression Analysis Based on Quantitative Real-Time PCR

We performed the qRT-PCR expression analysis for all the genes around the strongest associated loci with the capsule length (LMM) trait in order to pinpoint the potential candidate gene. Accession G330 with a long capsule size (~3.65 cm, at maturity stage) and accession G346 with a short capsule size (~1.90 cm, at maturity stage) were selected for this experiment. Capsules from the middle of the main stem were collected from 3 different plants (biological replicates) in Wuhan on 3, 6, 9, 12 and 21 days after pollination. RNA was extracted from fresh capsule tissues and reverse transcribed according to descriptions of Mmadi et al. [43]. In total, three genes were investigated and their gene-specific primers designed using the Primer5.0 tool [44] (Table S7). The qRT-PCR was conducted in triplicate (technical replicates) on a Roche Lightcyler^®^ 480 instrument (Roche Molecular Systems, Inc, Basel, Switzerland) using SYBR Green Master Mix (Vazyme), according to the manufacturer’s protocol. Reaction and PCR conditions are the same as the descriptions of Mmadi et al. [43]. The sesame *Actin* gene (*SIN_1006268*) was used as the internal reference and the relative gene expression values were calculated using the 2^−ΔCt^ method [45].

## Figures and Tables

**Table 1:** SNPs stably detected in different environments and for various traits.

LG	Position (bp)	Env.	Traits	PVE (%)	LG	Position (bp)	Env.	Traits	PVE (%)
1	1,700,170	SY	CNB	7.00	5	17,411,684	SY	SNMM	7.29
			CN	7.10				SWMM	6.79
1	1,994,183	SY	YB	8.68	6	3,404,764	SY	YB	10.65
			YMB	7.41				MCNB	7.33
1	4,450,107	SY	YB	7.55				YMB	11.15
			YMB	6.99	6	3,790,583	SY	TWB	7.13
1	6,149,415	SY	TMM	7.61				WMB	8.28
			WMM	6.58	6	5,995,560	SY	YB	10.91
1	8,185,969	SY	YB	7.03				CNB	6.99
			YMB	7.26				YMB	11.32
1	9,906,190	SY	YB	6.67	6	9,021,538	SY	YB	8.12
			YMB	6.88				YMB	8.34
1	11,118,941	SY	SNMB	6.32	6	9,971,772	NN	TMM	6.29
			SWMB	6.50				WMM	6.39
1	17,291,730	SY	YB	8.73	6	14,154,329	SY	YB	7.29
			YMB	9.10				YMB	6.59
2	1,201,448	SY	YB	8.76	6	14,581,641	SY	LCNM	10.29
			YMB	9.15				CNM	6.41
			YB	6.41	6	14,701,957	WH	TMM	7.09
2	5,260,400	SY	YB	7.08				WMM	6.96
			YMB	7.87	6	15,551,496	SY	RMM	7.63
2	6,057,670	NN	TMM	9.46				SNMM	6.13
			WMM	11.33	6	21,992,131	SY	YB	10.89
2	7,236,995	SY	YB	6.09				YMB	10.77
			YMB	6.11	7	6,763,527	SY	RMB	6.69
2	8,388,879	NN	TWB	6.32				SWMB	7.65
			TWB	7.24	7	1,702,826	NN	CNM	9.42
2	9,244,103	SY	TMM	6.04			WH	CNM	6.66
		WH	WMM	9.85	8	1,398,196	SY	YB	11.12
			TMM	9.25				YMB	11.99
2	11,245,765	SY	TMM	7.95	8	1,668,572	SY	YB	7.38
			WMM	7.56				SNMM	9.89
2	15,016,082	SY	YB	7.51				YM	6.66
			YMB	7.78				SWMM	8.84
2	17,451,873	SY	SNMB	6.47	8	21,325,953	SY	YB	8.97
			RMM	9.17				YMM	7.68
			SWMM	14.89	9	1,007,867	SY	RMM	8.57
			SWMB	6.83				SNMM	8.58
			SNMM	15.13	9	954,526	WH	SNMM	7.48
3	4,840,197	SY	LCNM	6.12				WMM	8.03
			CNM	6.42				TMM	7.80
3	13,198,513	SY	YB	6.68	10	1,647,805	SY	CNB	12.64
			YMB	7.24				CN	6.14
3	14,990,430	SY	YB	8.68	10	3,823,922	SY	CNB	7.43
			CNB	9.34				MCNB	6.85
			MCNB	9.40	10	7,418,158	NN	TMM	6.24
			YMB	8.81				WMM	6.31
3	16,939,689	SY	YB	6.70	10	8,305,398	SY	YB	7.76
			YMB	6.63				YMB	7.89
3	20,410,997	SY	TMM	6.47	10	10,792,029	SY	YB	7.29
			WMM	6.79				YMB	6.60
3	20,876,555	SY	YB	10.76	10	12,008,065	SY	RMM	6.83
			YMB	11.09				SNMM	6.61
3	20,878,243	SY	CNB	8.06	10	14,650,964	SY	YB	6.81
			MCNB	8.44				YMM	7.50
3	24,164,350	SY	TMM	6.84	10	15,097,365	SY	TMM	7.41
			WMM	6.43				WMM	6.90
4	2,505,014	SY	YB	7.98	11	6,996,833	SY	SNMM	6.53
			YMB	7.82				SWMM	7.78
4	6,419,408	SY	WMM	6.99	11	11,923,935	SY	YB	7.10
			CPMM	6.25				YMB	7.77
4	14,211,075	SY	YB	7.47	11	14,876,966	SY	YB	6.33
			YMB	6.10				YMB	6.91
5	202,984	SY	YB	9.50	11	15,137,600	SY	TMM	7.52
			YMM	6.92				WMM	6.55
5	2,854,336	NN	YM	7.85	12	328,609	SY	YM	7.53
			CNM	8.20				YMM	7.78
5	5,479,753	SY	YB	7.61	12	2,356,955	SY	YB	6.24
			CNB	8.89				YMB	6.72
			YMB	7.81	12	4,200,237	SY	YB	6.60
			CNM	13.68				YMB	7.21
		NN	CNM	17.90	12	4,895,688	SY	SNMM	8.67
5	6,737,753	SY	CNB	8.89				SWMM	6.18
			CNM	13.68	13	2,772,629	SY	YB	8.17
		NN	CNM	17.90				YMB	8.49
5	6,738,735	NN	LCNM	7.01	14	194,410	SY	YB	6.20
								YMB	6.70
			CNM	13.30	15	2,174,040	SY	YB	7.20
		SY	LCNM	13.03				YMB	7.22
		WH	CNM	14.79	15	2,372,143	WH	YB	6.29
5	6,757,688	NN	SNMB	8.02				CNM	7.03
			SWMB	10.88				YM	6.01
5	9,869,746	NN	RMB	6.54	15	3,989,016	SY	YB	6.72
			SWMB	6.08				YMB	6.69
5	11,806,702	SY	TWB	6.29	16	555,771	WH	TMM	7.55
			TWB	6.34				WMM	6.40
5	15,855,382	WH	SNMM	6.76	16	1,633,469	SY	YB	7.97
			TMM	9.11				YMB	8.17
			WMM	8.68	16	2,989,809	SY	CNB	9.26
		NN	WMM	6.23				MCNB	8.26
5	17,340,920	SY	CNB	8.64					
			MCNB	10.45					

LG = Linkage group; Env. = Environment; PVE = Phenotypic variance explained; SY = Sanya; NN = Nanning; WH = Wuhan.

**Table 2:** Shared genomic regions detected for seed yield-related traits between our GWAS results and previous linkage mapping QTLs.

Traits Linkage Mapping	Code	LG	Start (bp)	End (bp)	Traits GWAS	LG	SNP Position (bp)
Grain number per capsule	Qgn-6	6	1,739,987	2,125,872	YB	6	1,741,236
					YLB	6	2,081,828
Capsule number per plant	Qcn-11	9	6,032,193	8,312,219	MCNM	9	5,988,865
					CNB	9	7,589,997
					MCNB	9	7,839,050
Capsule length	Qcl-3	3	1,566,853	2,593,783	YB	3	2,588,239
					YMB	3	2,588,241
	Qcl-4	5	9,840,981	10,961,395	YLB	5	9,857,730
					RMB	5	9,869,746
					SWMB	5	9,869,746
					TMM	5	9,895,178
					WMB	5	9,974,401
					TMB	5	10,197,769
					TMB	5	10,208,013
					YLB	5	10,705,889
					SWMB	5	10,773,145
					WMB	5	10,781,532
					LLM	5	10,786,506
					CN	5	10,786,597
					LCNM	5	10,790,853
					SWLM	5	10,958,834
	Qcl-8	4	11,220,208	11,670,895	LCNM	4	11,649,295
					WMM	4	11,658,278
					TSW	4	11,661,092
	Qcl-12	11	14,935,946	15,400,039	LLM	11	14,957,580
					LLM	11	15,003,280
					TMM	11	15,137,600
					WMM	11	15,137,600
					CPMM	11	15,138,140
					LLM	11	15,200,435
					LMM	11	15,219,964
					LMM	11	15,239,947
					LMM	11	15,289,738

## Supplementary Materials

Supplementary materials can be found at http://www.mdpi.com/1422-0067/19/9/2794/s1. Figure S1. Boxplots displaying variation of 33 traits across three different environments (SY = Sanya, NN = Nanning and WH = Wuhan). Figure S2. Manhattan plots for SNP association of all traits in the three environments (SY = Sanya, NN = Nanning and WH = Wuhan). Table S1. Full name of the 39 assayed traits. Table S2. Summary of descriptive statistics of the 39 traits in three environments. Table S3. List and position of the significant loci detected in this study. Table S4. List and functional annotation of genes around peak loci associated with the assayed traits in this study. Table S5. Candidate gene-containing significant SNPs detected in this study and their homologs in *Arabidopsis thaliana*. Table S6. Full list of the 705 accessions used in this study, their origin and their breeding status. Table S7. Primer sequences for qRT-PCR gene expression analysis.

## Abbreviations

CN	effective capsule number in plant
CNB	effective capsule number in branch
CNM	effective capsule number in main stem
CPLM	dry capsule pericarp weight of lateral capsule in main stem
CPMB	dry capsule pericarp weight of medial capsule in branch
CPMM	dry capsule pericarp weight of medial capsule in main stem
DNA	deoxyribonucleic acid
GWAS	genome wide association study
LCNB	effective lateral capsule number in branch
LCNM	effective lateral capsule number in main stem
LD	linkage disequilibrium
LG	linkage group
LLB	length of lateral capsule in branch
LLM	length of lateral capsule in main stem
LMB	length of medial capsule in branch
LMM	length of medial capsule in main stem
MAF	minor allele frequency
MCNB	effective medial capsule number in branch
MCNM	effective medial capsule number in main stem
NN	Nanning
qRT-PCR	quantitative real-time polymerase chain reaction
QTL	quantitative trait loci
RLM	ratio of seed weight and capsule pericarp weight for lateral capsule in main stem
RMB	ratio of seed weight and capsule pericarp weight for medial capsule in branch
RMM	ratio of seed weight and capsule pericarp weight for medial capsule in main stem
RNA	ribonucleic acid
SNLB	seed number per lateral capsule in branch
SNLM	seed number per lateral capsule in main stem
SNMB	seed number per medial capsule in branch
SNMM	seed number per medial capsule in main stem
SNP	single nucleotide polymorphism
SY	Sanya
SWLB	dry seed weight of per lateral capsule in branch
SWLM	dry seed weight of per lateral capsule in main stem
SWMB	dry seed weight of per medial capsule in branch
SWMM	dry seed weight of per medial capsule in main stem
TLM	thickness of lateral capsule in main stem
TMB	thickness of medial capsule in branch
TMM	thickness of medial capsule in main stem
TSW	thousand seeds weight
WH	Wuhan
WLM	width of lateral capsule in main stem
WMB	width of medial capsule in branch
WMM	width of medial capsule in main stem
YB	yield of branch per plant
YLB	yield of lateral capsules in branch
YLM	yield of lateral capsules in main stem
YM	yield of main stem per plant
YMB	yield of medial capsules in branch
YMM	yield of medial capsules in main stem
YP	yield per plant
