# Supplementary material for: Genome-Wide Association Studies of 39 Seed Yield-Related Traits in Sesame (Sesamum indicum L.)
Source: Int J Mol Sci. 2018 Sep 17;19(9):2794. doi: 10.3390/ijms19092794 (PMC6164633; doi:10.3390/ijms19092794)

LCNM-SY

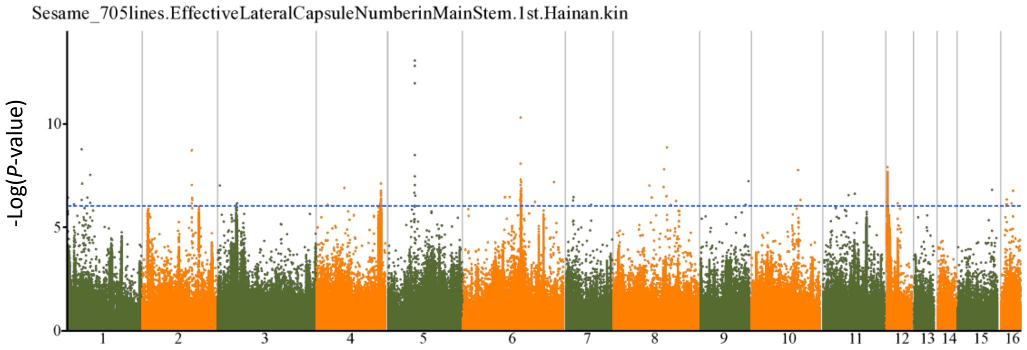

LCNM-NN

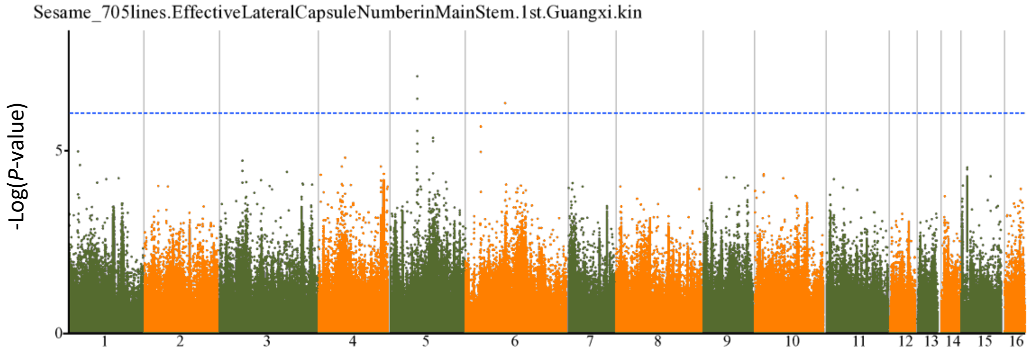

LCNM-WH

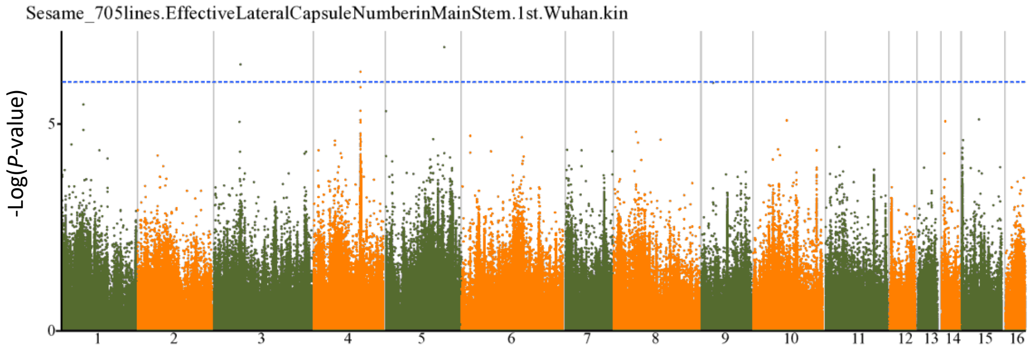

CPMB-SY

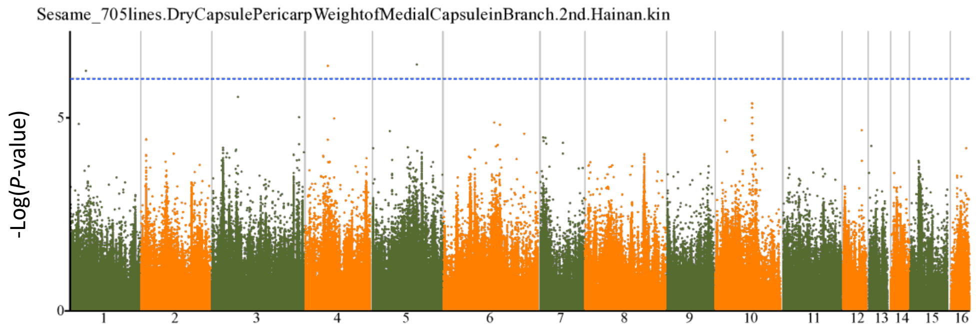

CPMB-NN

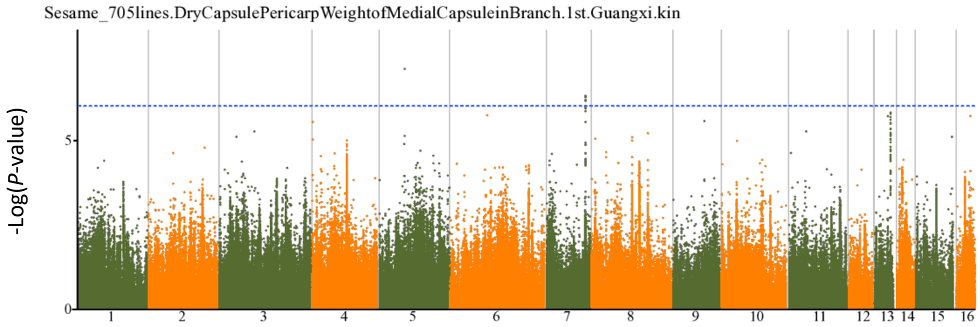

CPMB-WH

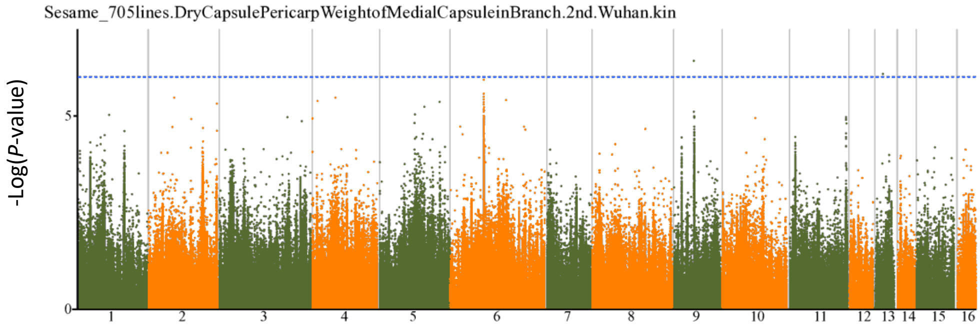

CPLM-SY

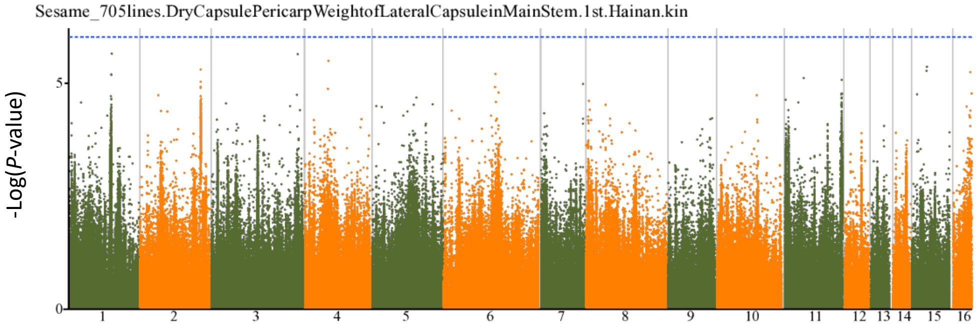

CPLM-NN

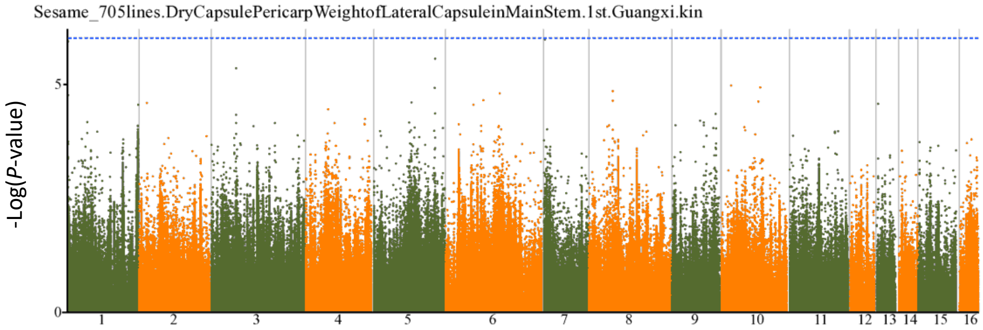

CPLM-WH

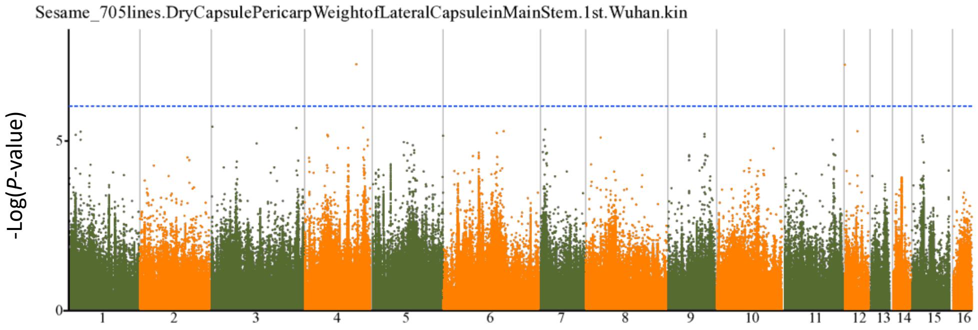

CPMM-SY

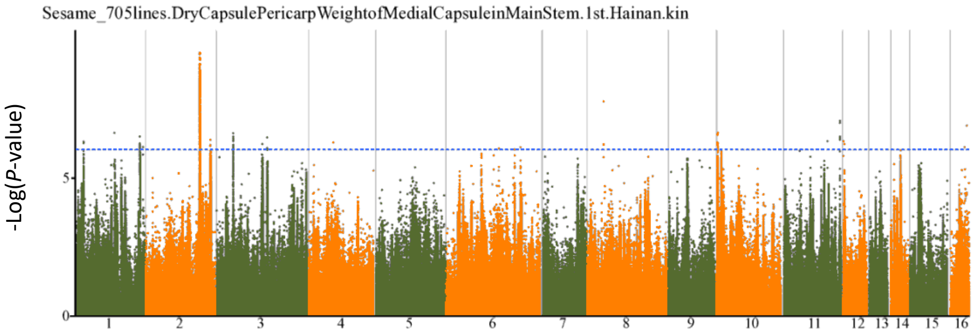

CPMM-NN

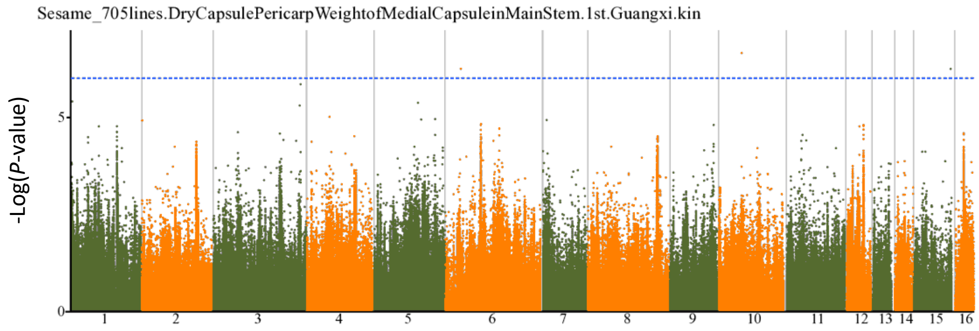

CPMM-WH

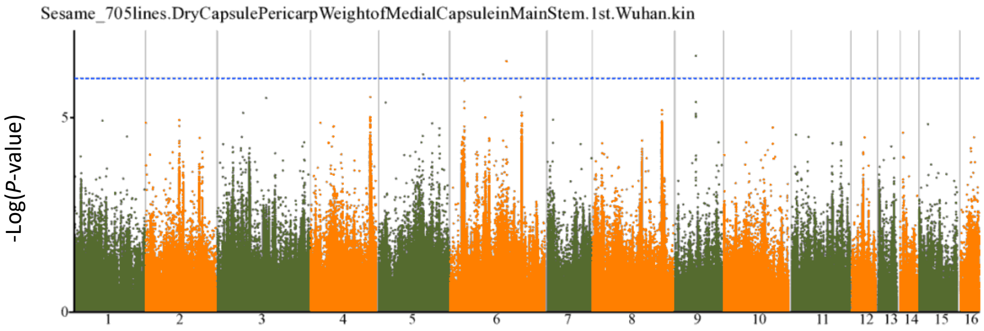

SWLB-SY

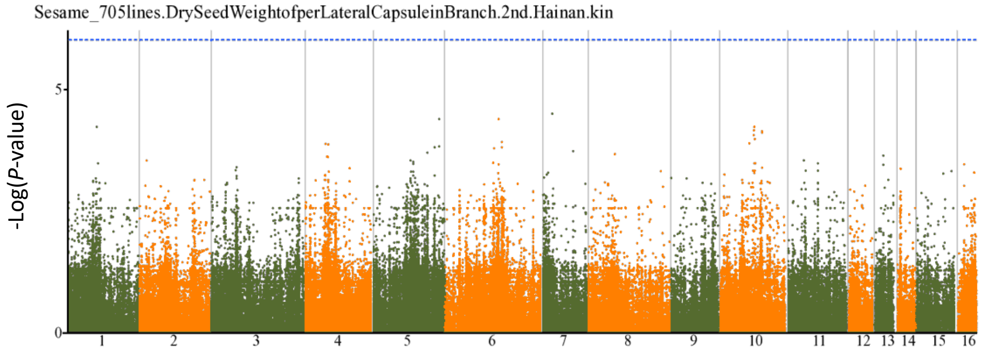

SWLB-NN

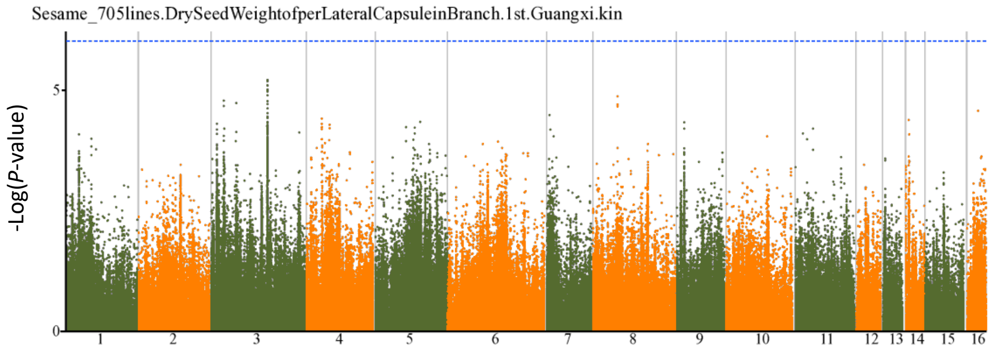

SWLB-WH

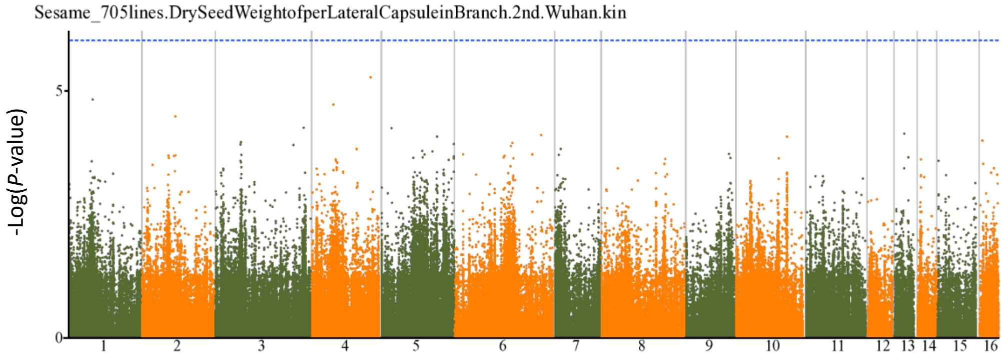

SWLM-SY

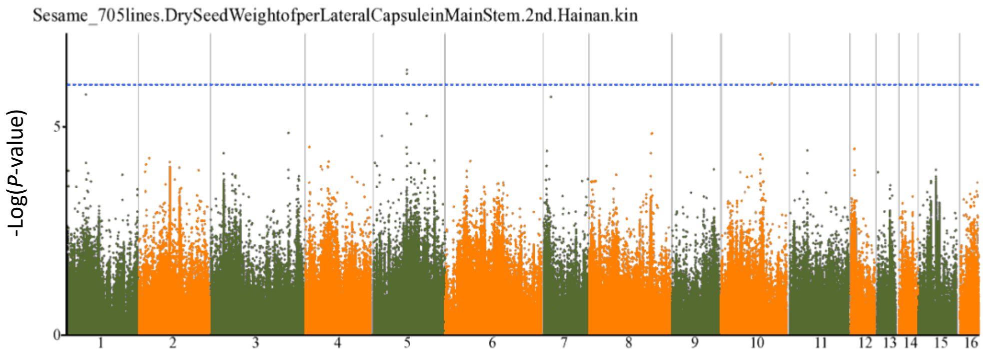

SWLM-NN

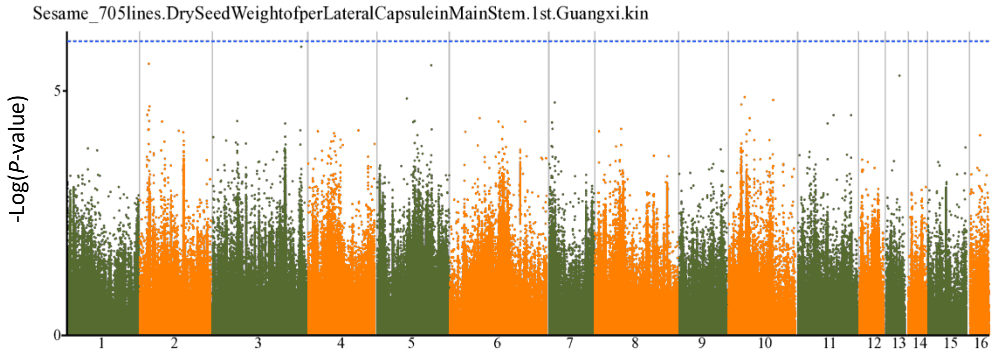

SWLM-WH

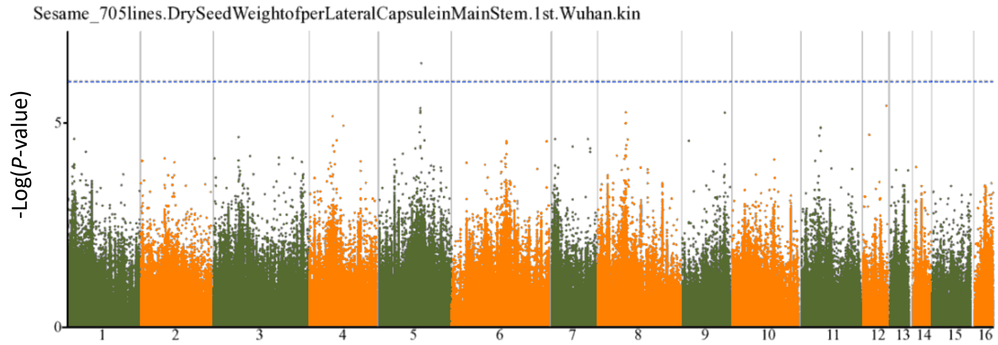

SWMB-SY

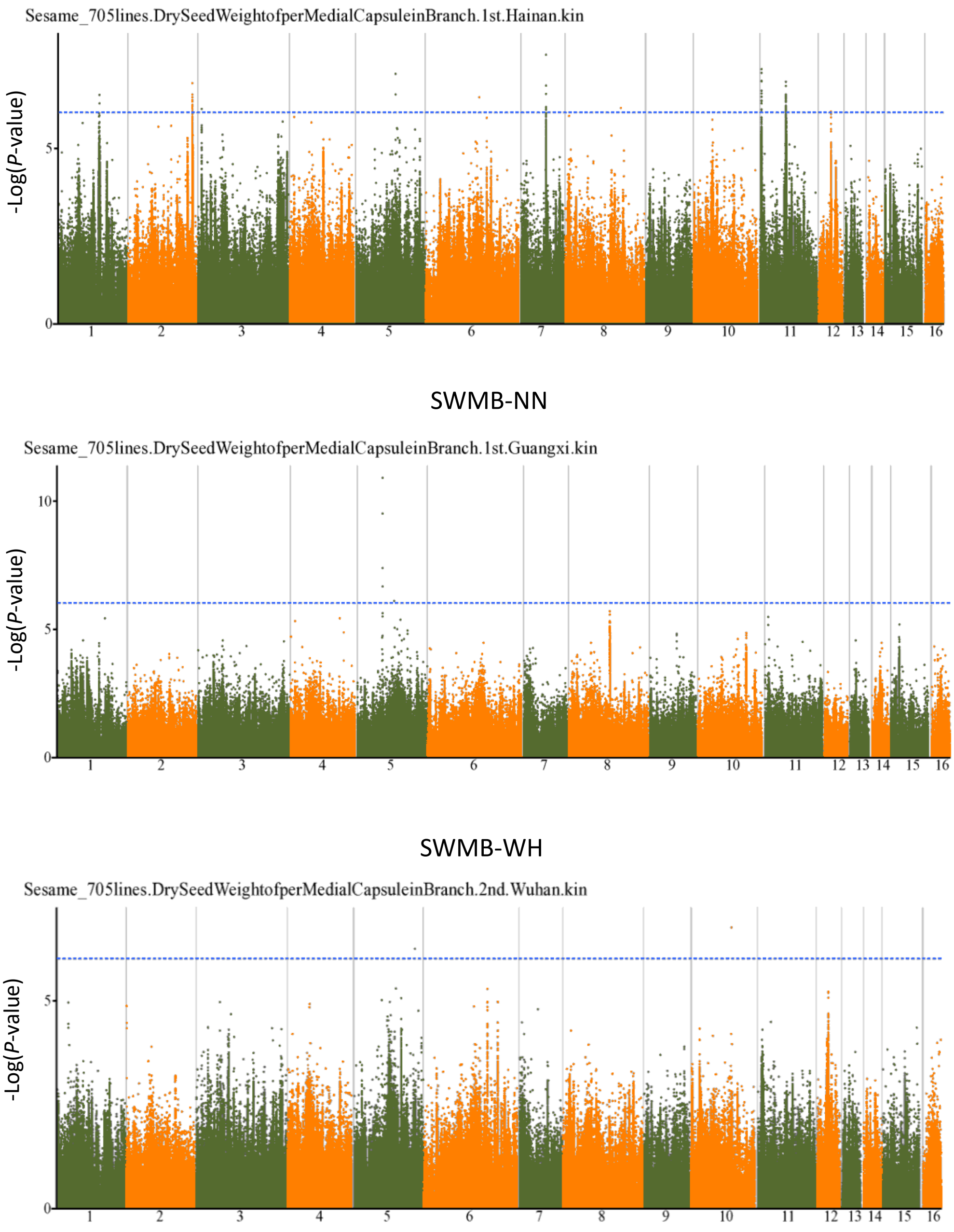

SWMB-NN

SWMB-WH

SWMM-SY

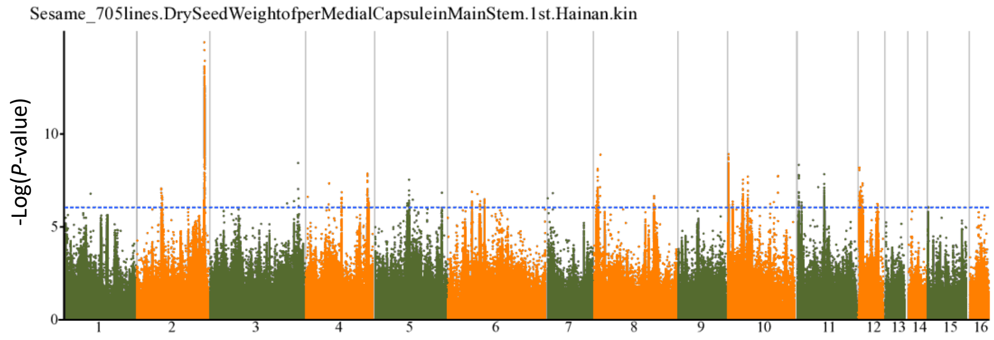

SWMM-NN

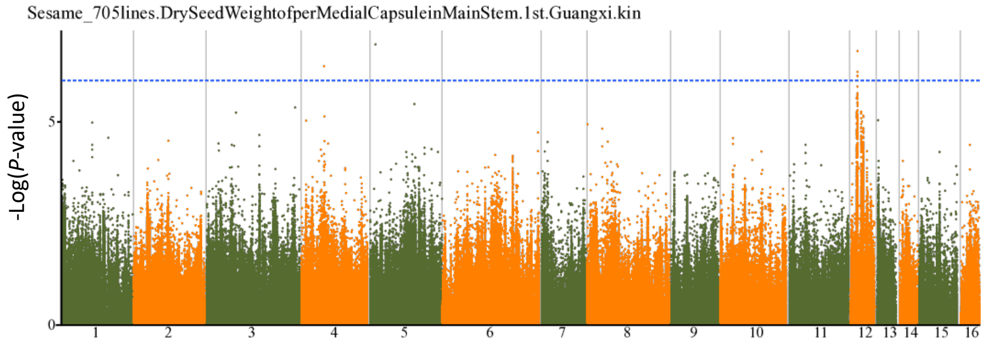

SWMM-WH

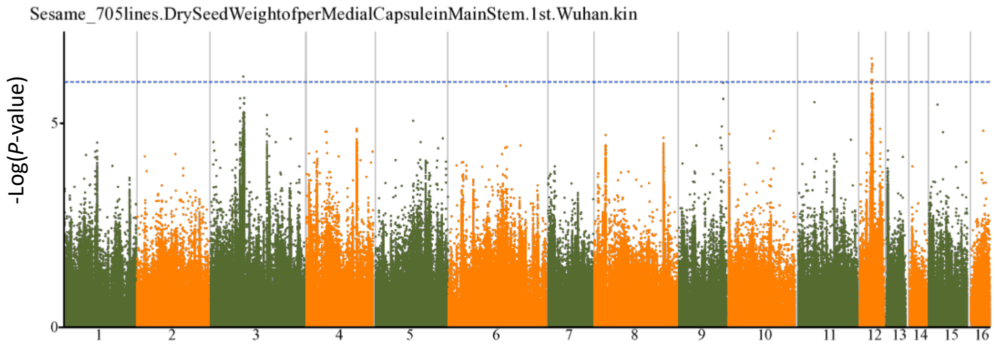

CNB-SY

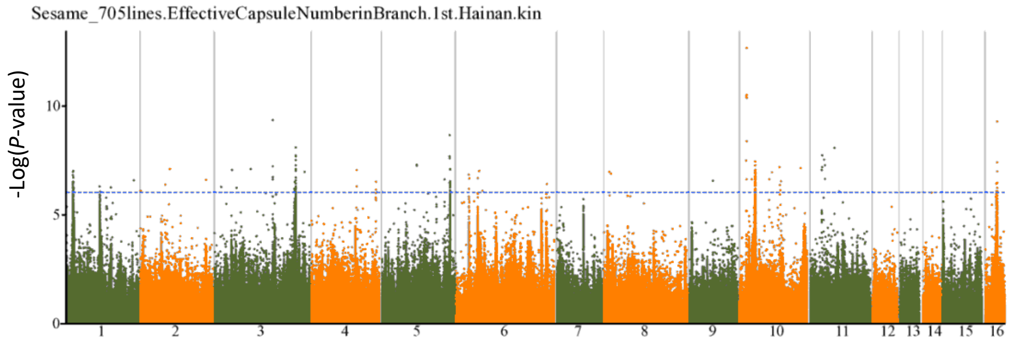

CNB-NN

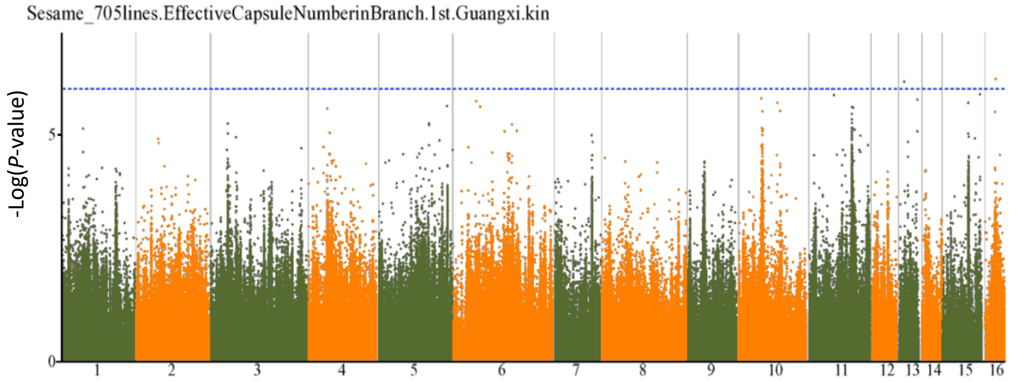

CNB-WH

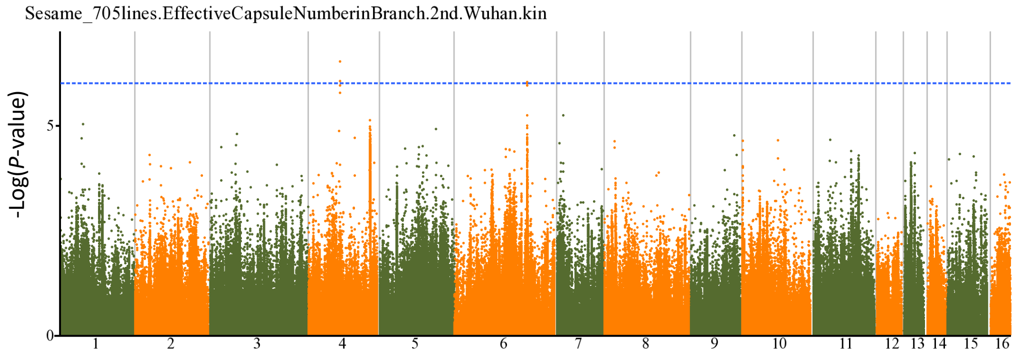

CN-SY

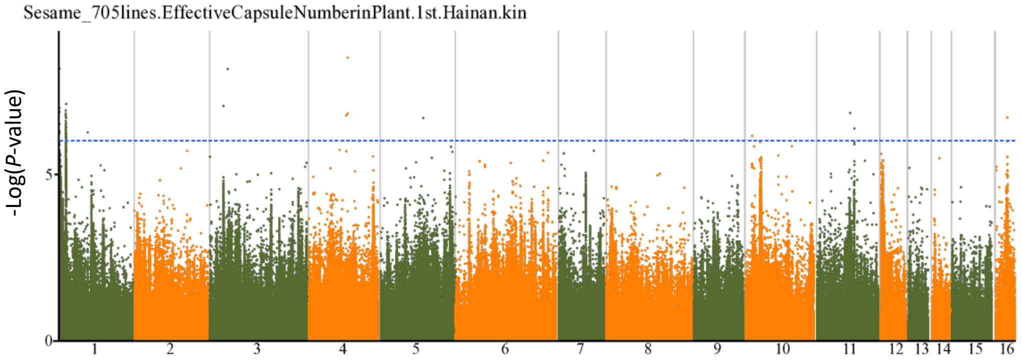

CN-NN

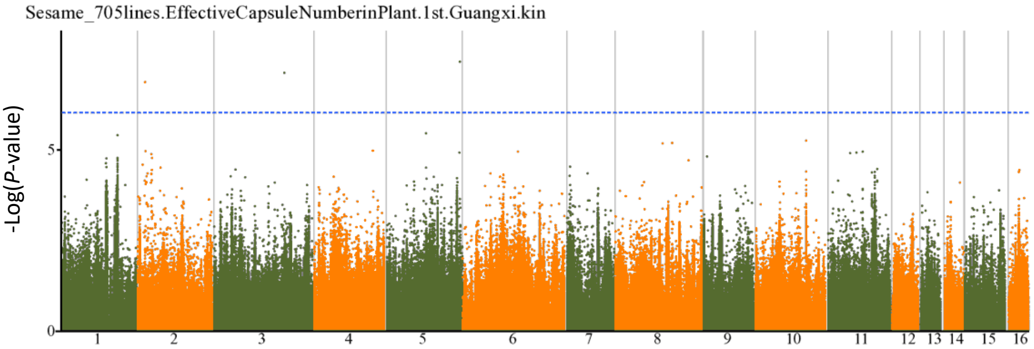

CN-WH

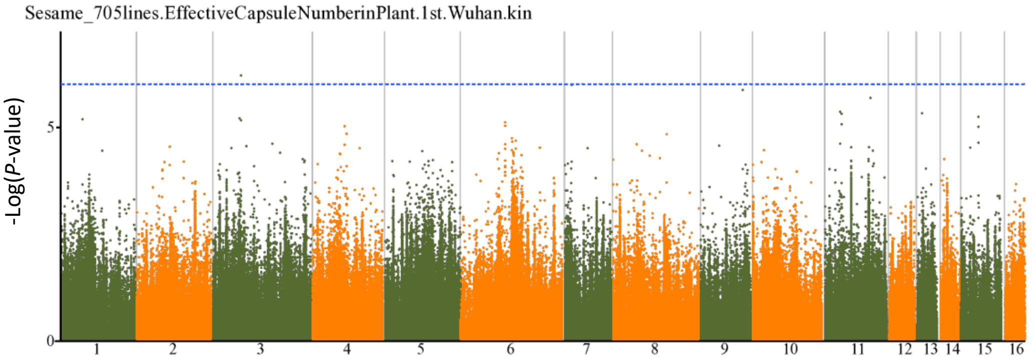

LCNB-SY

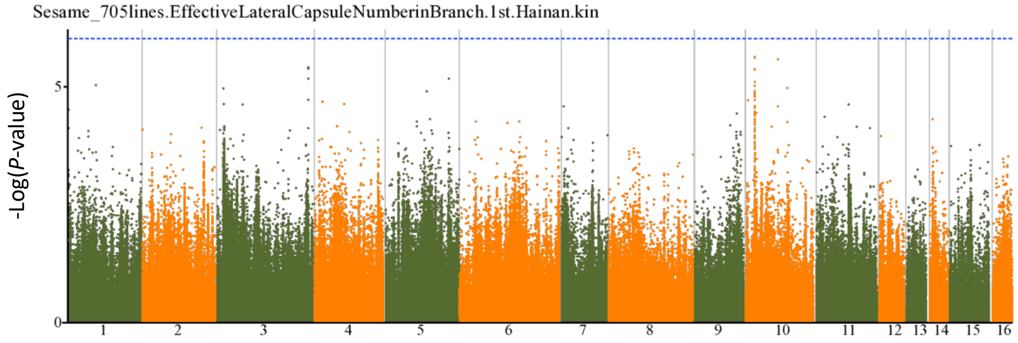

LCNB-NN

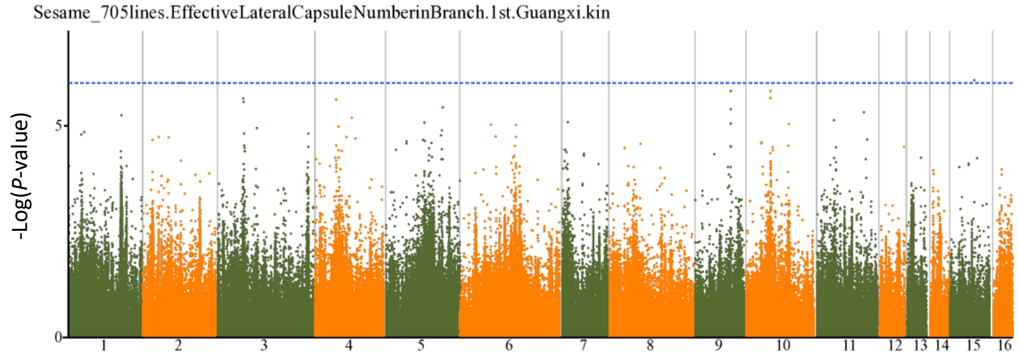

LCNB-WH

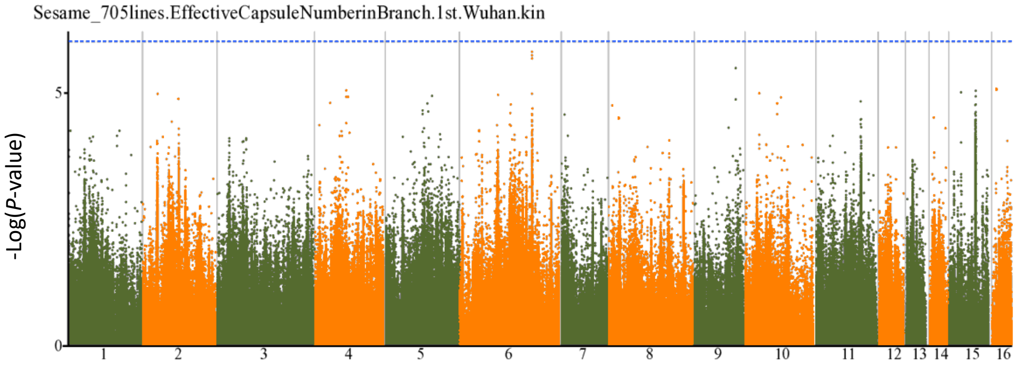

MCNB-SY

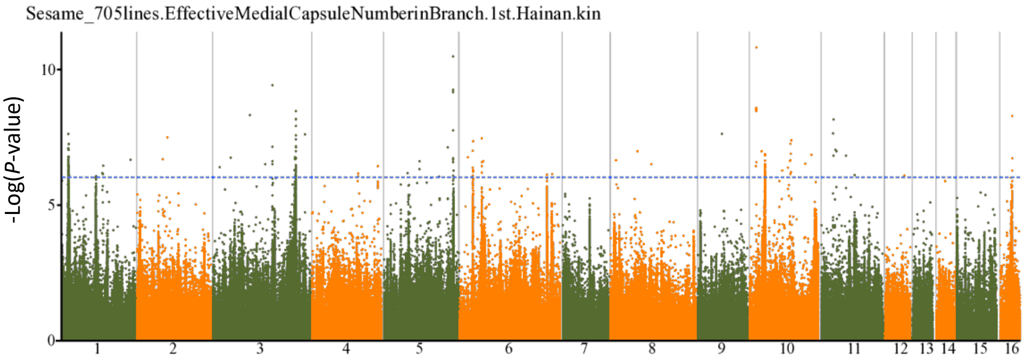

MCNB-NN

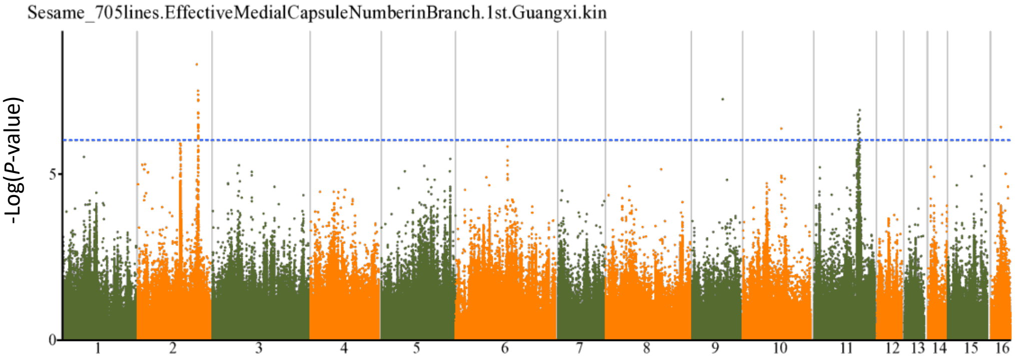

MCNB-WH

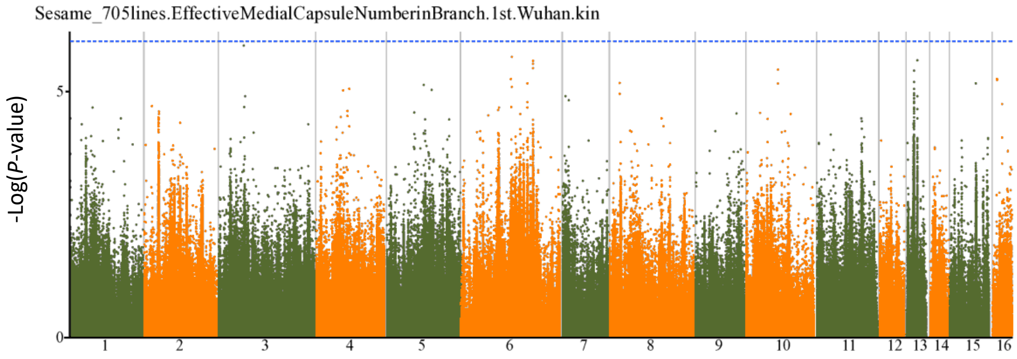

MCNM-SY

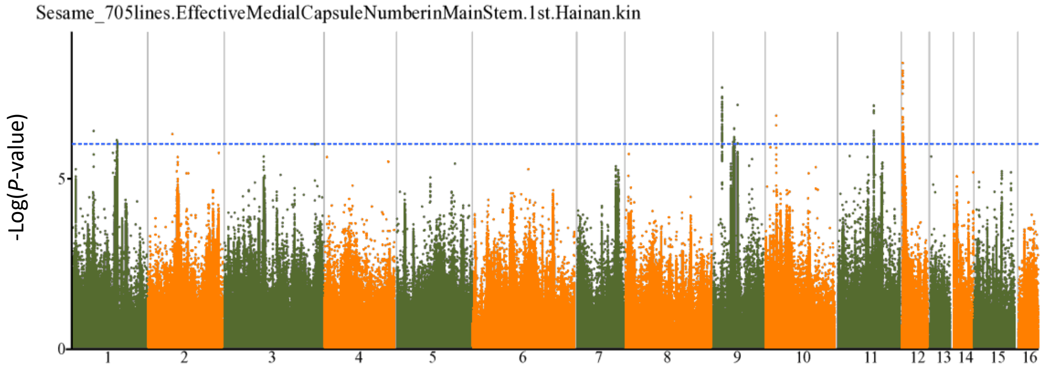

MCNM-NN

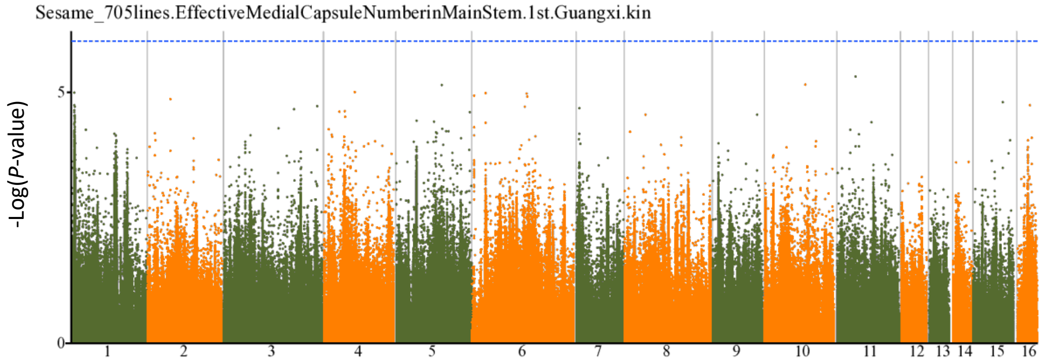

MCNM-WH

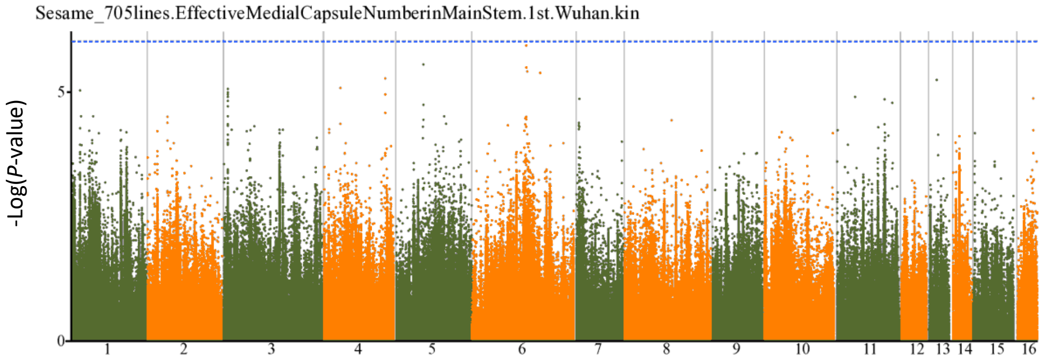

LLB-SY

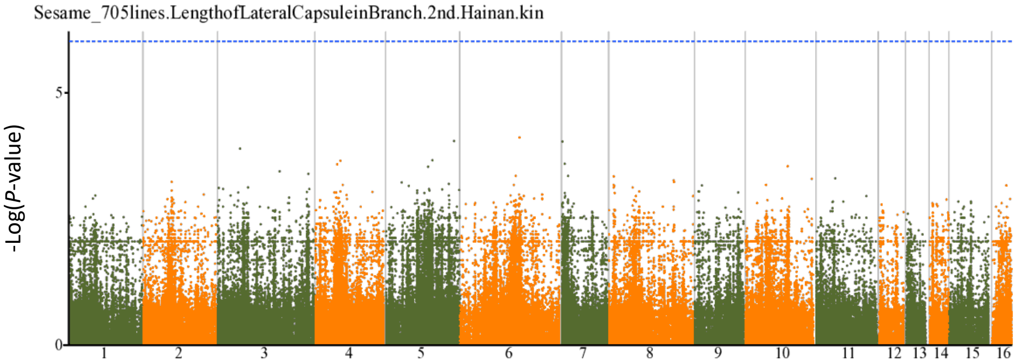

LLB-NN

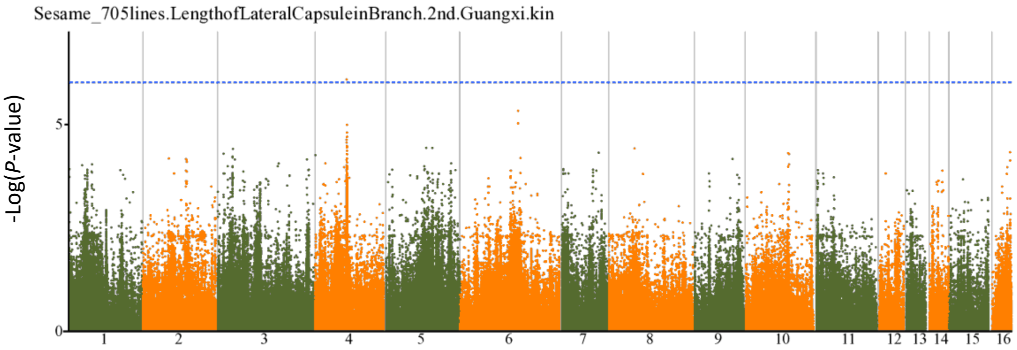

LLB-WH

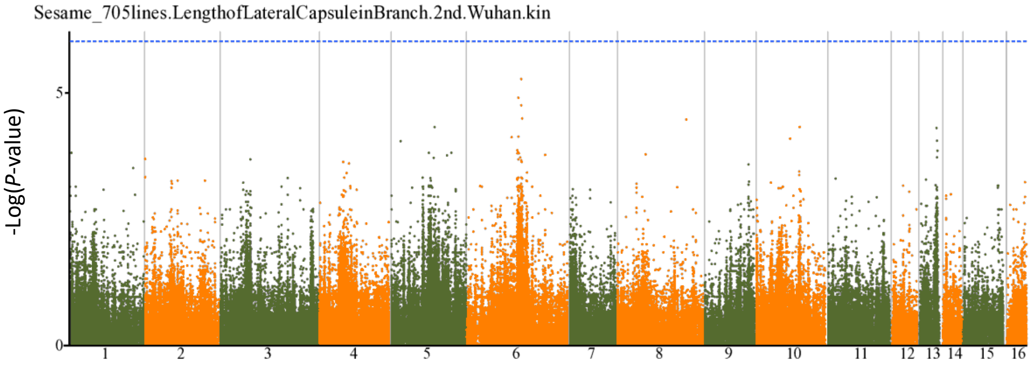

LLM-SY

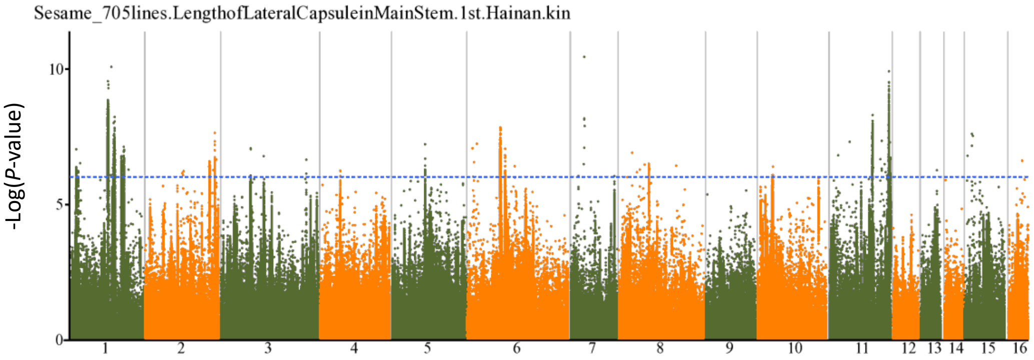

LLM-NN

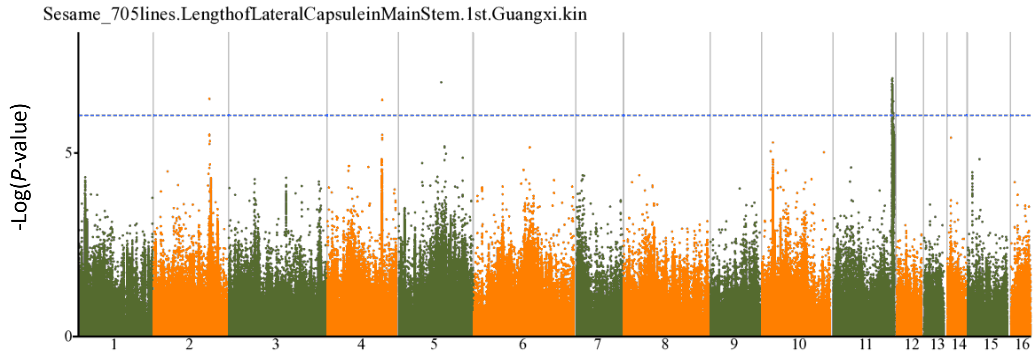

LLM-WH

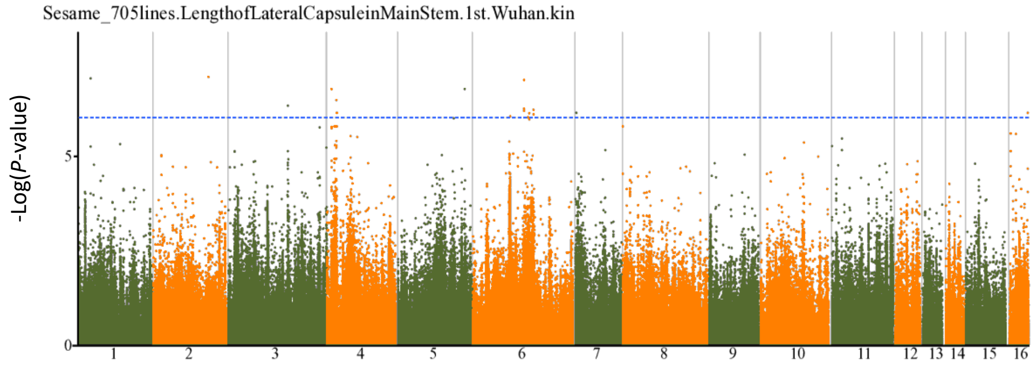

LMB-SY

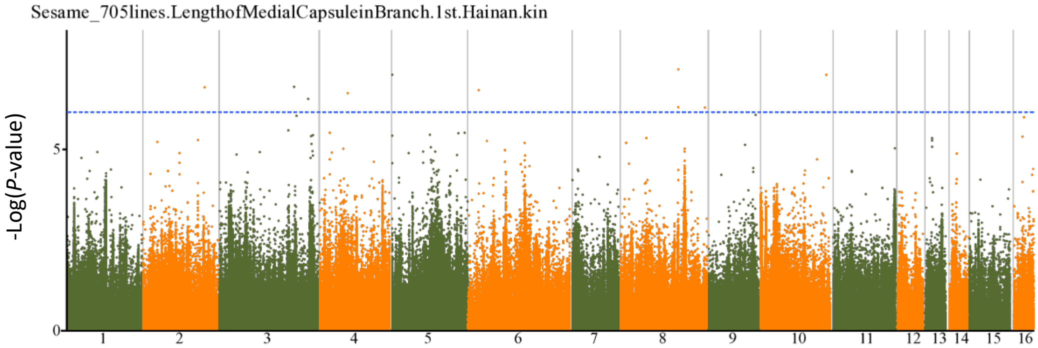

LMB-NN

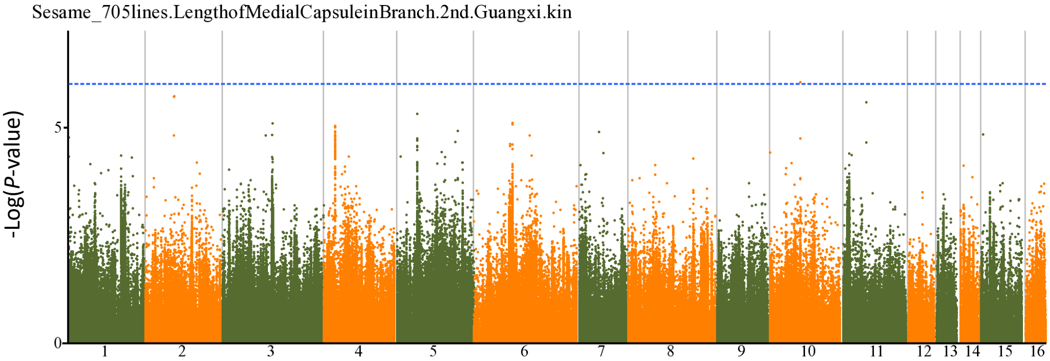

LMB-WH

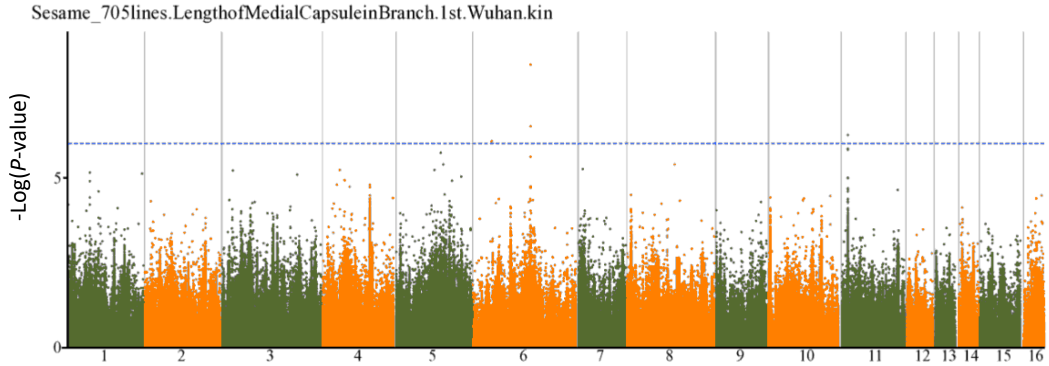

RLM-SY

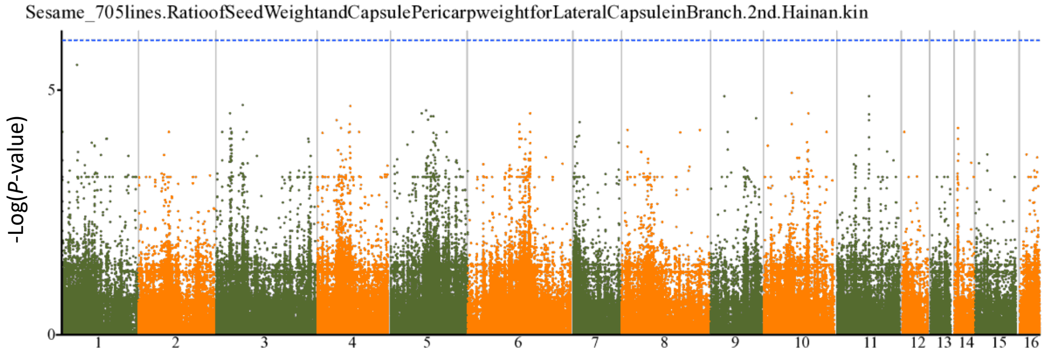

RLM-NN

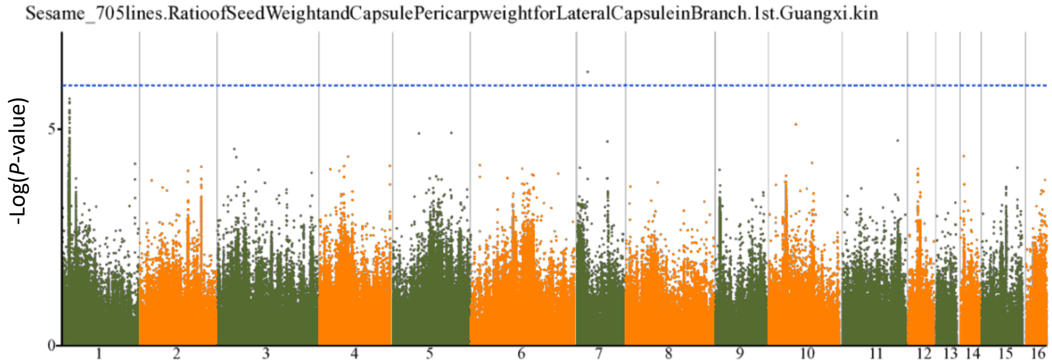

RLM-WH

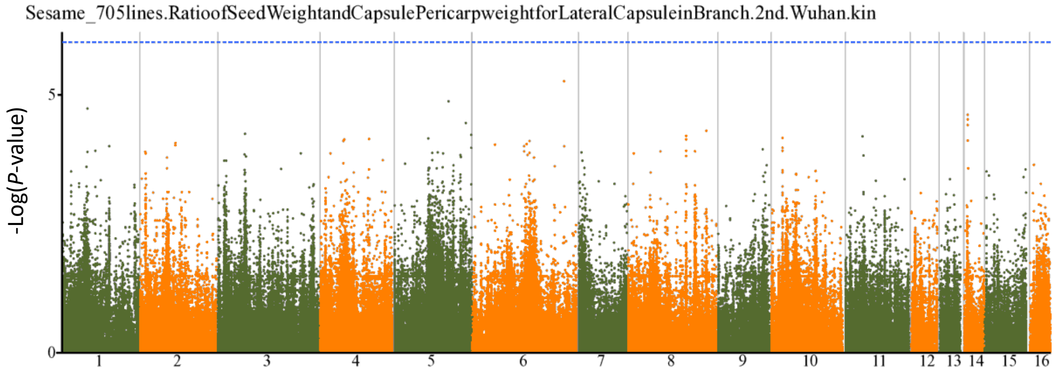

RMB-SY

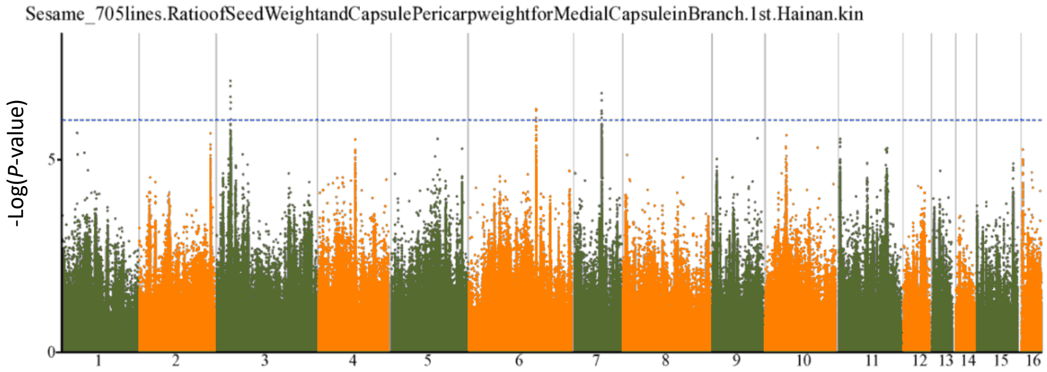

RMB-NN

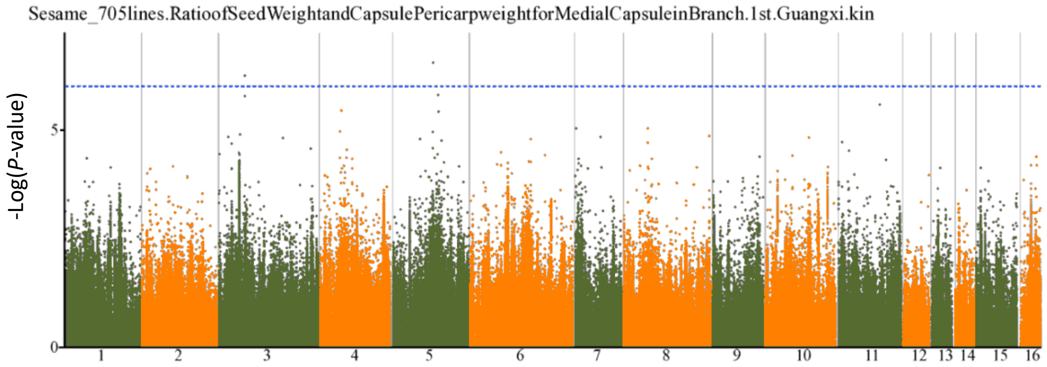

RMB-WH

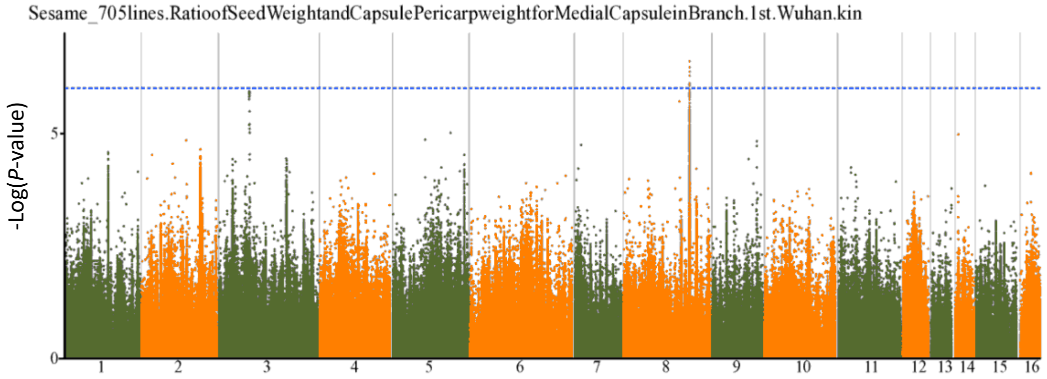

RMM-SY

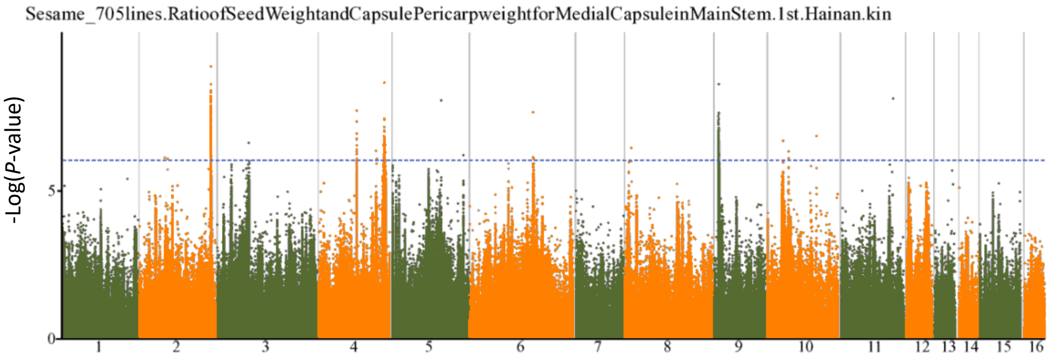

RMM-NN

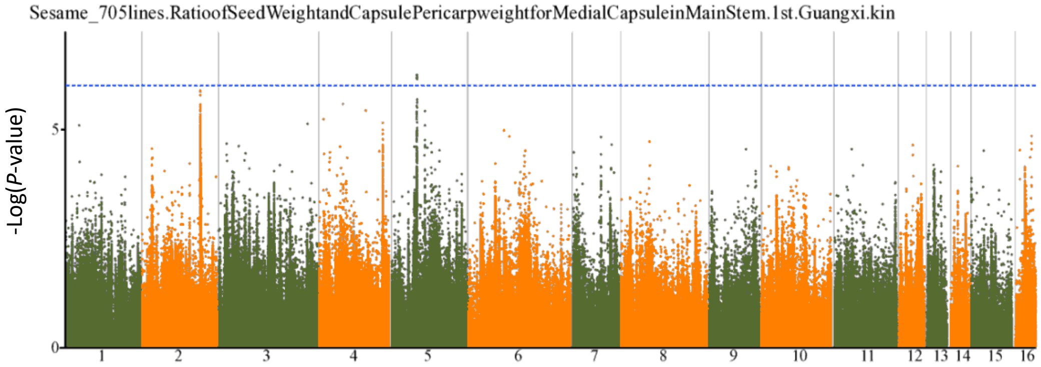

RMM-WH

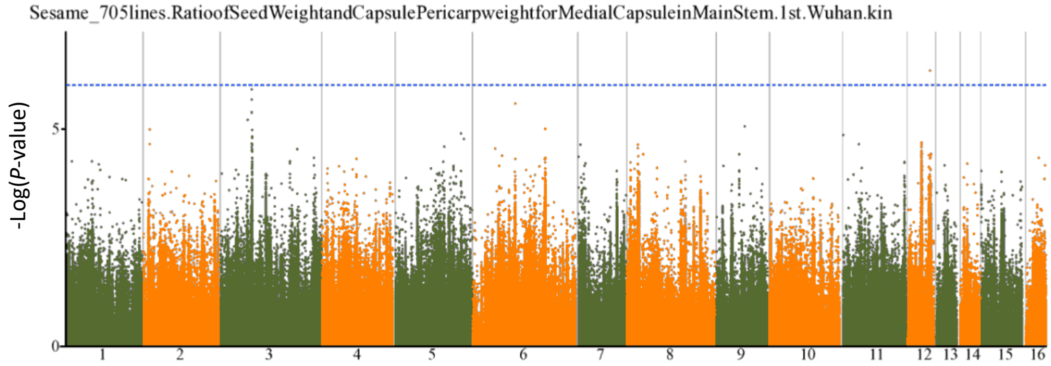

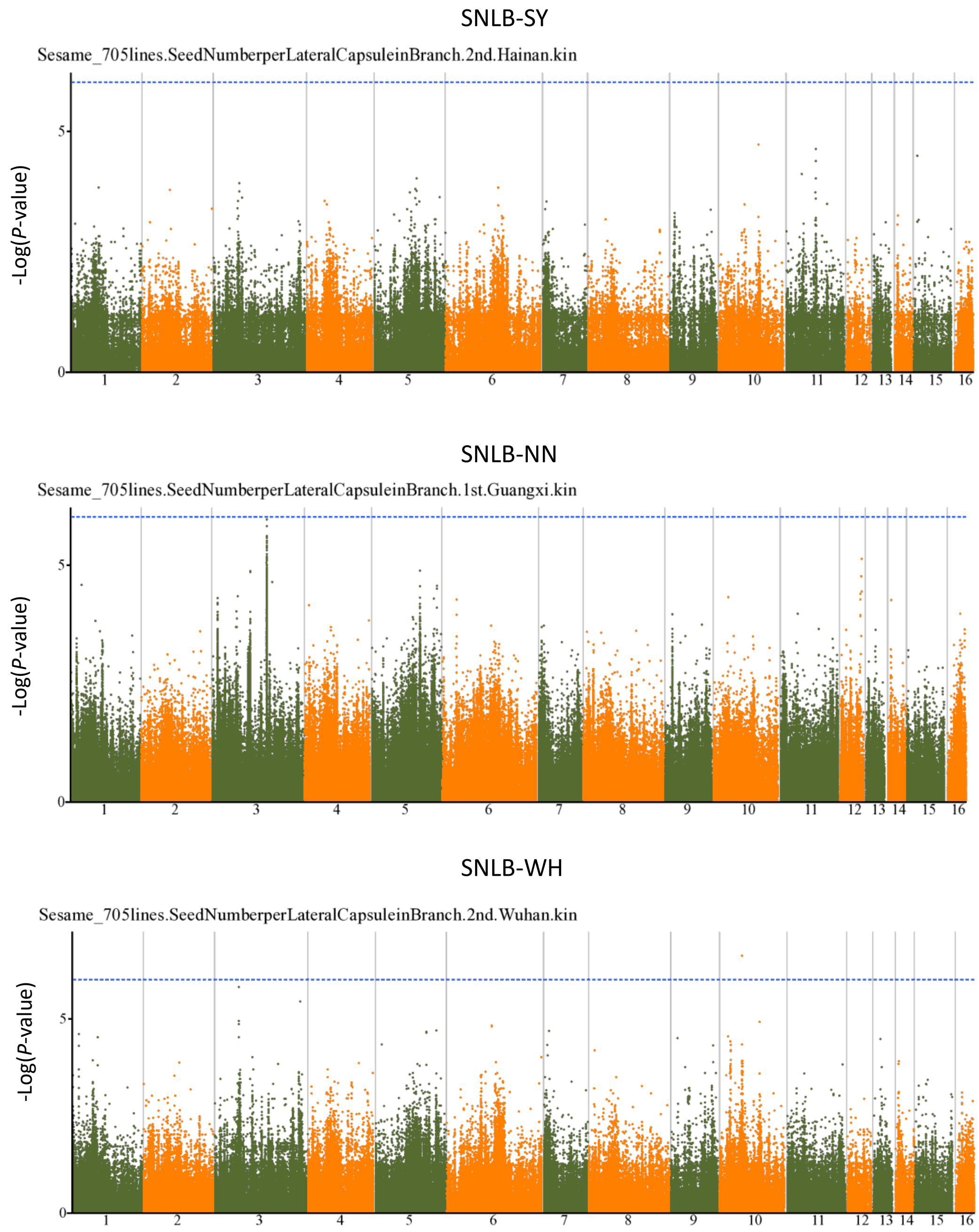

SNLB-SY
Sesame_705lines.SeedNumberperLateralCapsuleinBranch.2nd.Hainan.kin
-Log(P-value)
SNLB-NN
Sesame_705lines.SeedNumberperLateralCapsuleinBranch.1st.Guangxi.kin
-Log(P-value)
SNLB-WH
Sesame_705lines.SeedNumberperLateralCapsuleinBranch.2nd.Wuhan.kin
-Log(P-value)

SNLM-SY

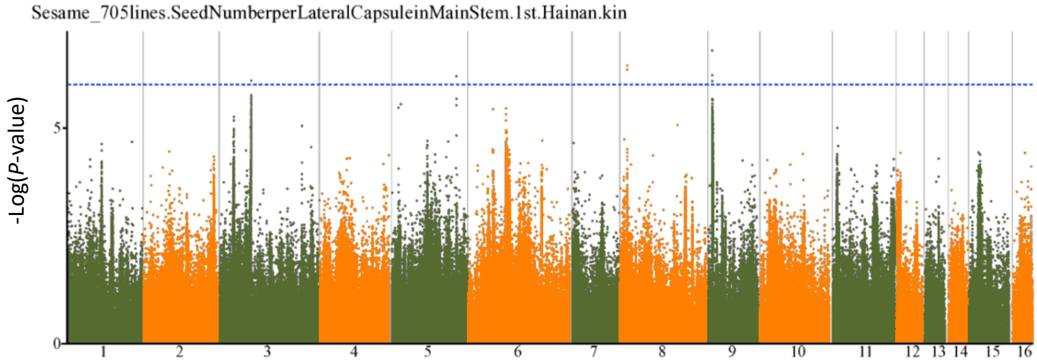

SNLM-NN

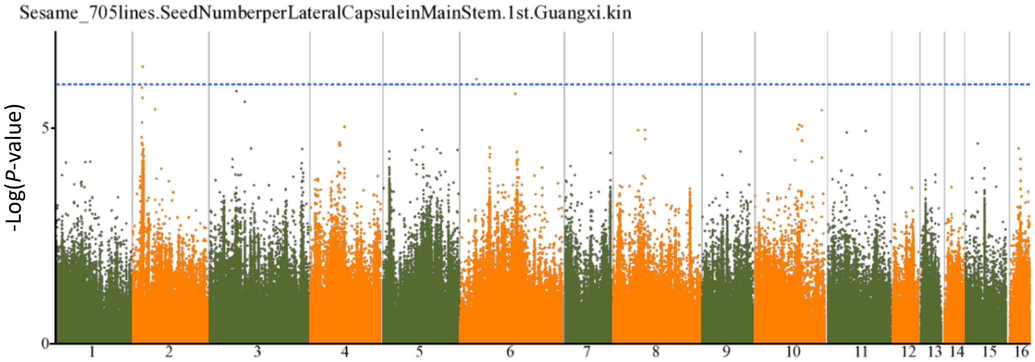

SNLM-WH

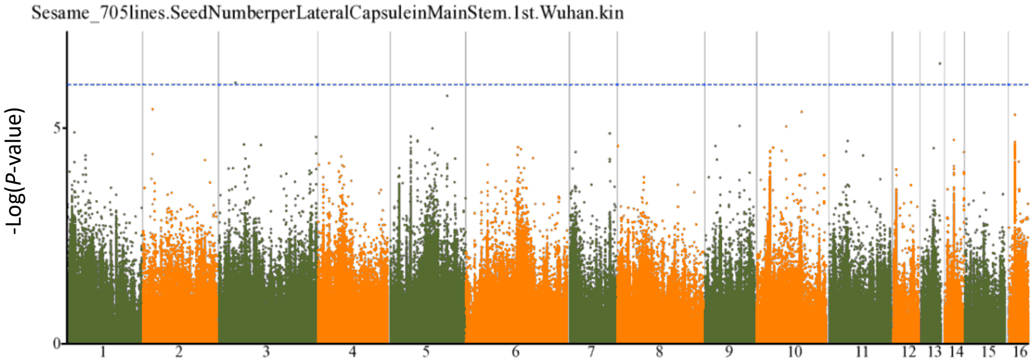

SNMB-SY

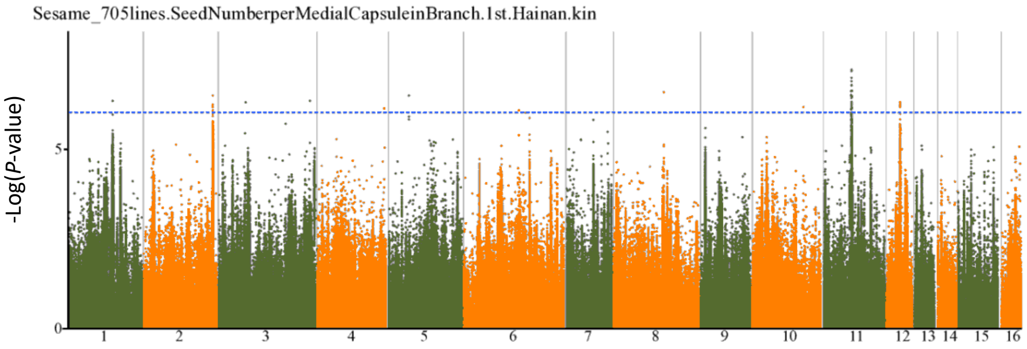

SNMB-NN

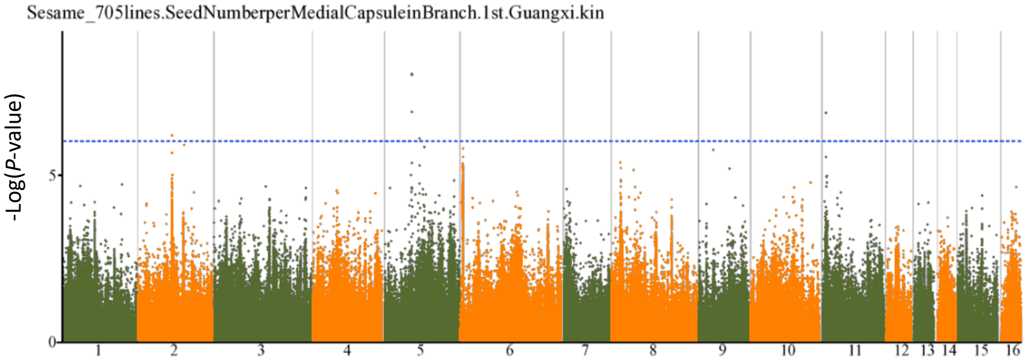

SNMB-WH

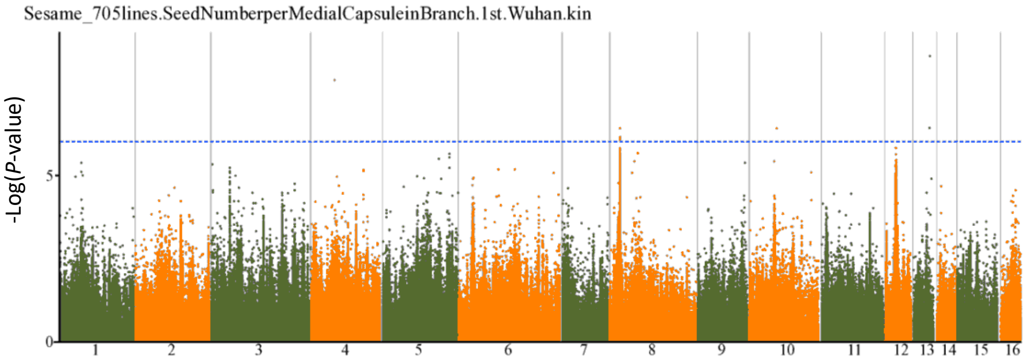

SNMM-SY

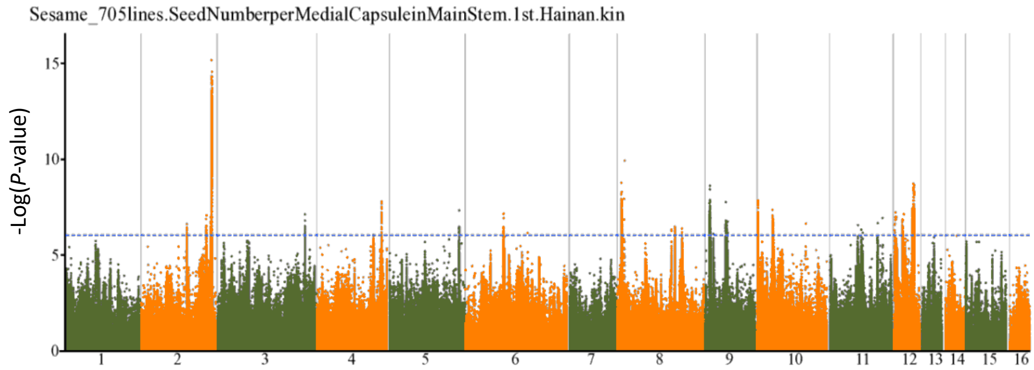

SNMM-NN

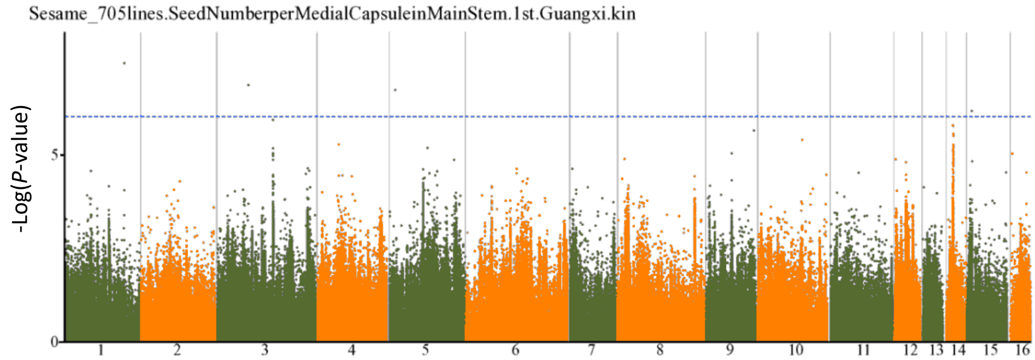

SNMM-WH

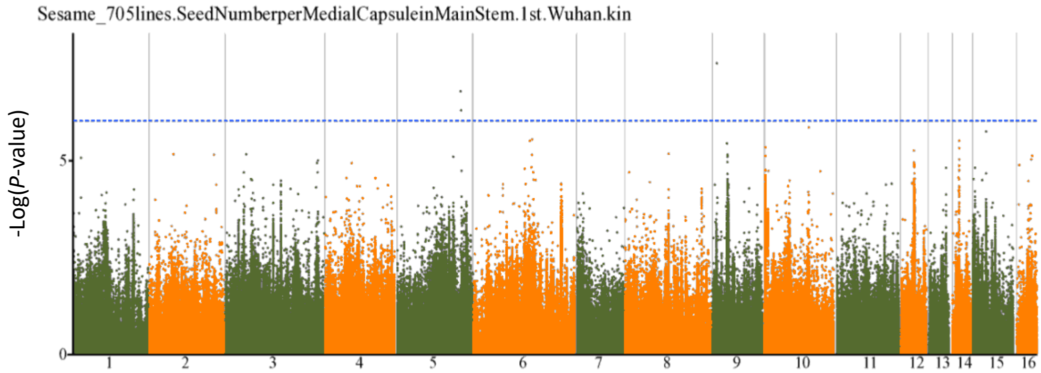

TLM-SY

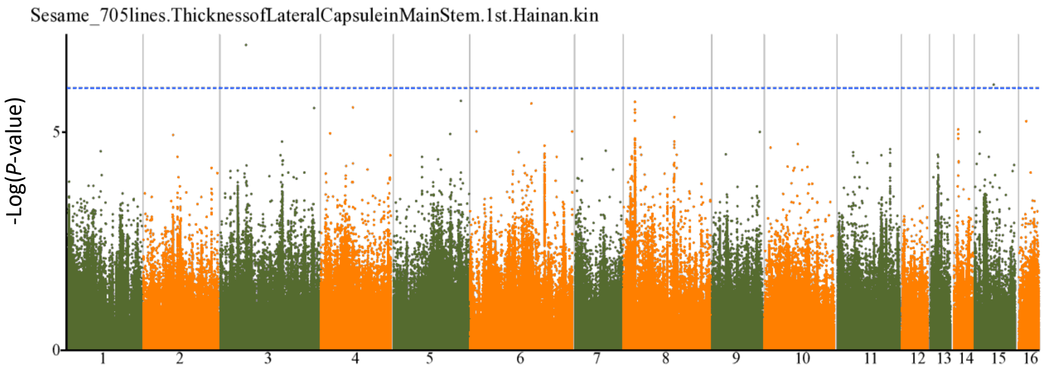

TLM-NN

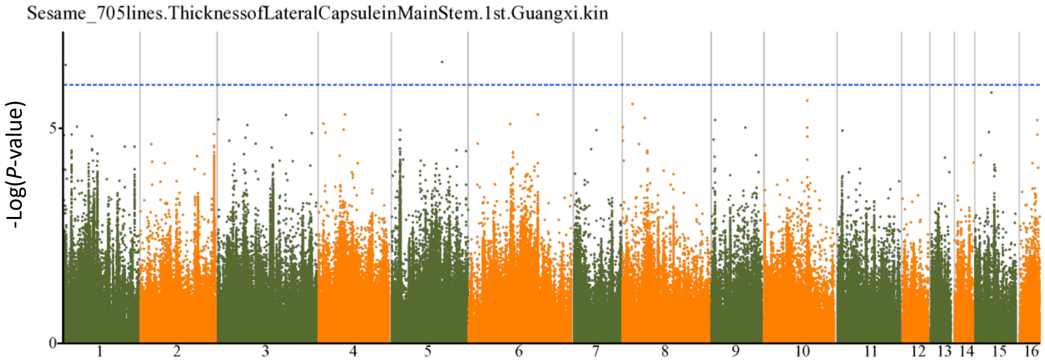

TLM-WH

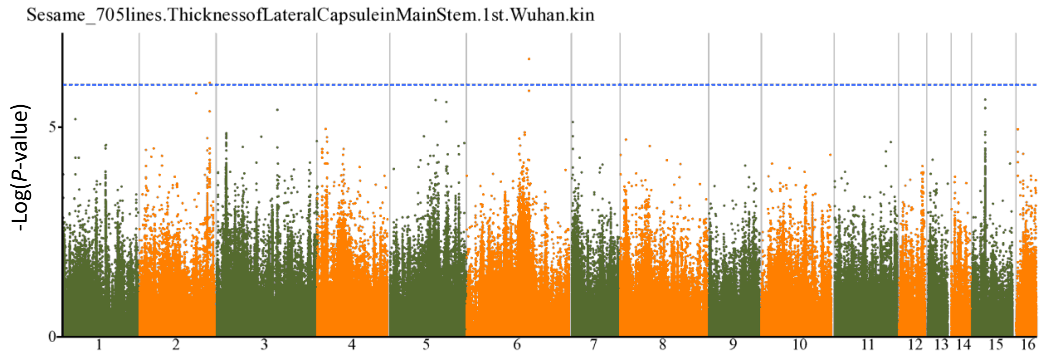

TMB-SY

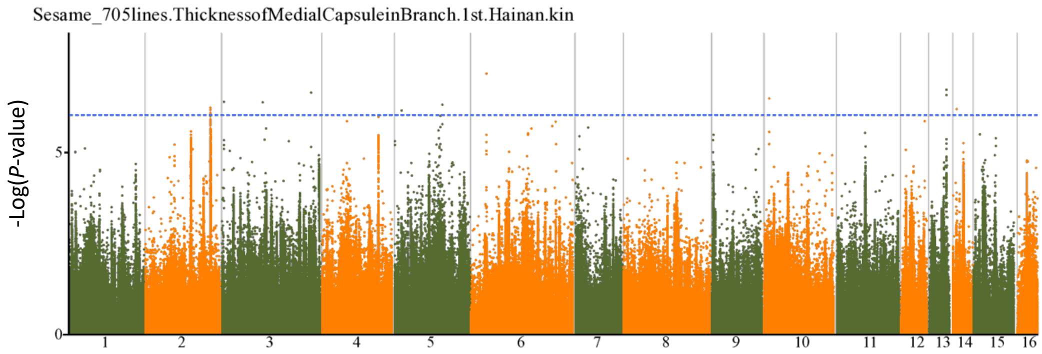

TMB-NN

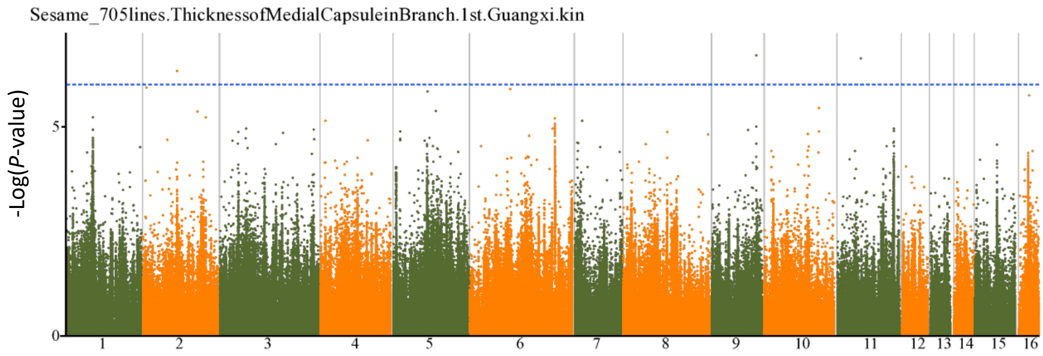

TMB-WH

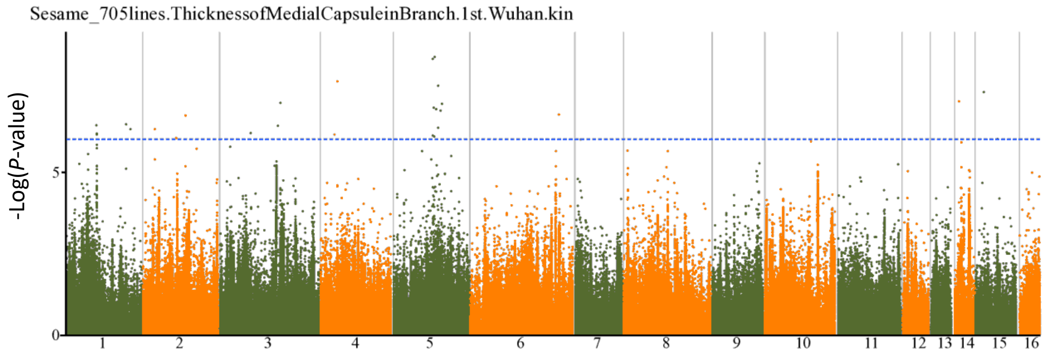

TMM-SY

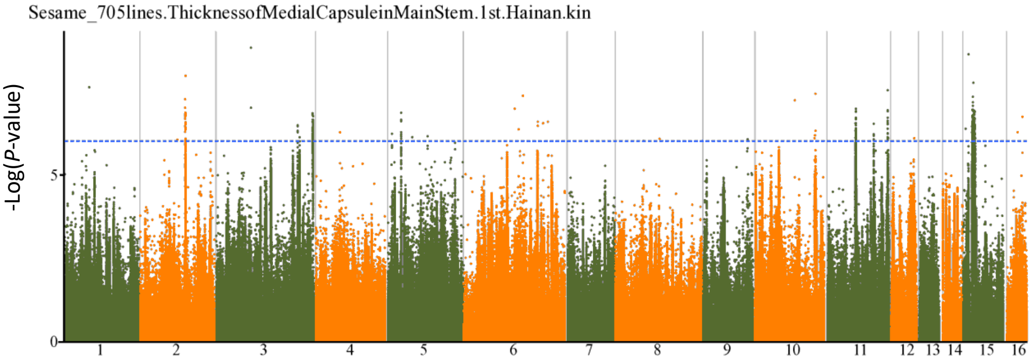

TMM-NN

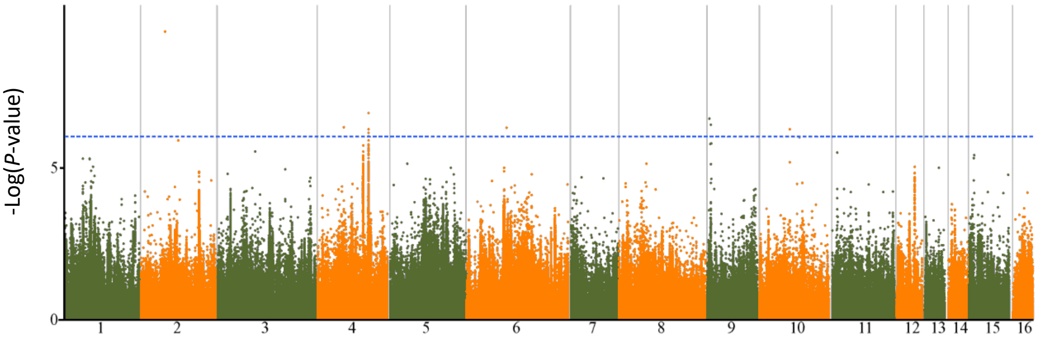

TMM-WH

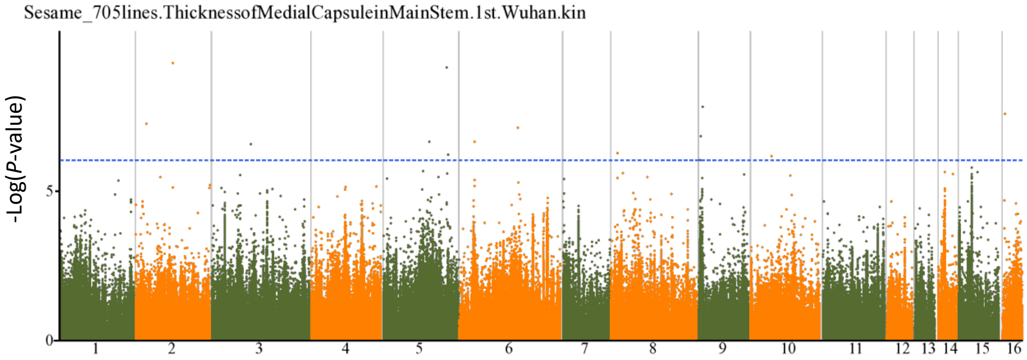

TSW-NN

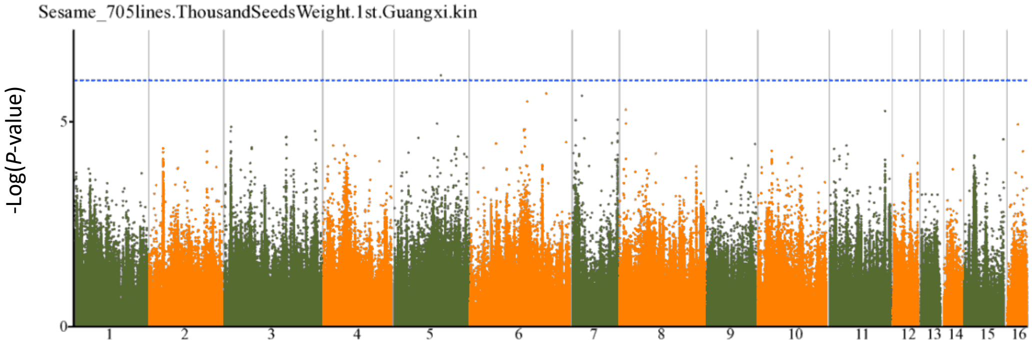
Sesame_705lines.ThousandSeedsWeight.1st.Guangxi.kin
-Log(P-value)
5
0
1
2
3
4
5
6
7
8
9
10
11
12
13
14
15
16

TSW-WH

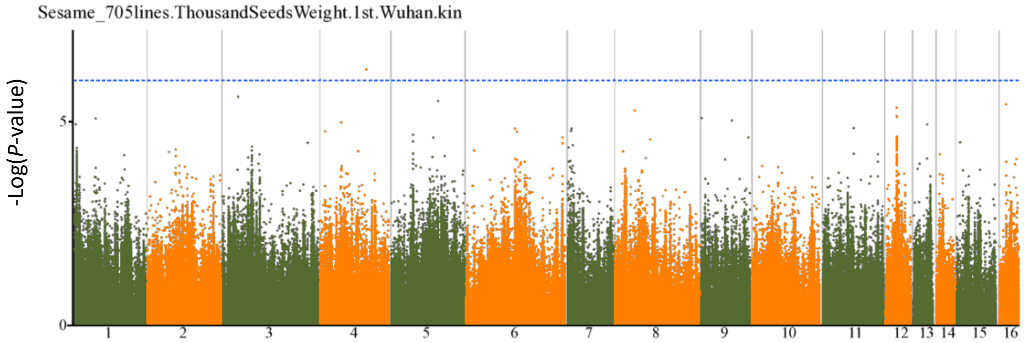
Sesame_705lines.ThousandSeedsWeight.1st.Wuhan.kin
-Log(P-value)
5
0
1
2
3
4
5
6
7
8
9
10
11
12
13
14
15
16

WLM-SY

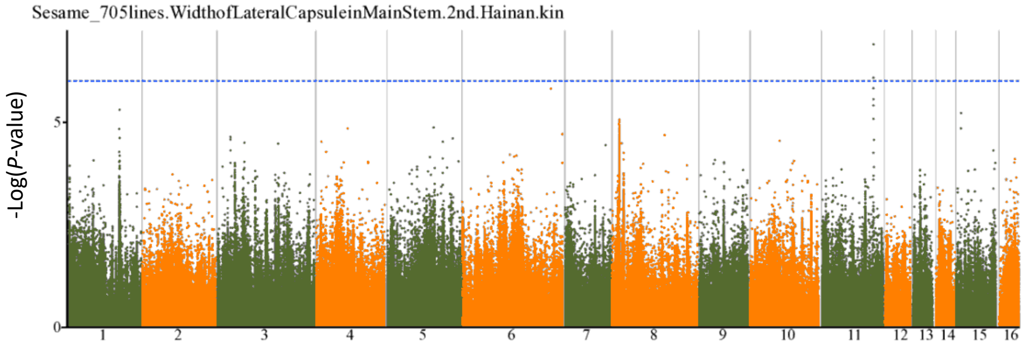

WLM-NN

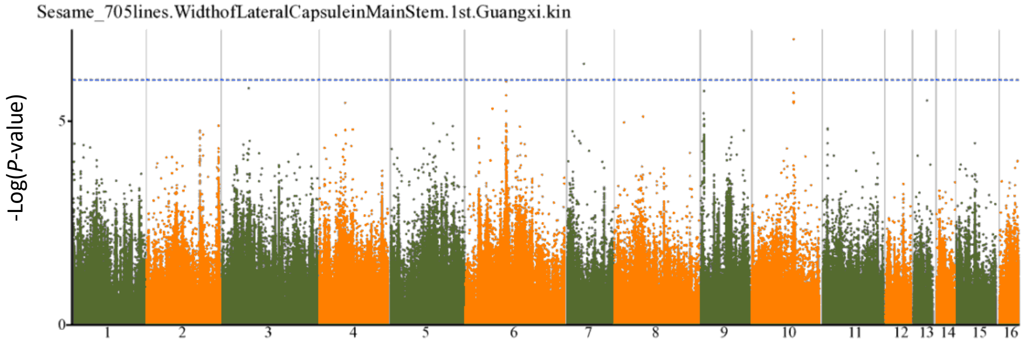

WLM-WH

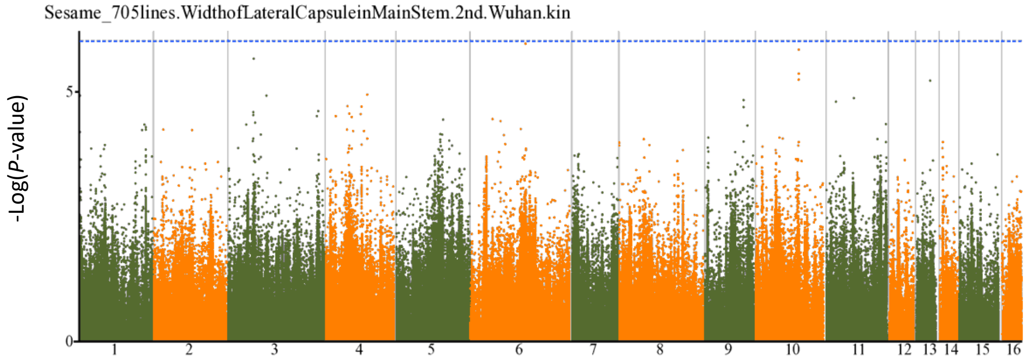

WMB-SY

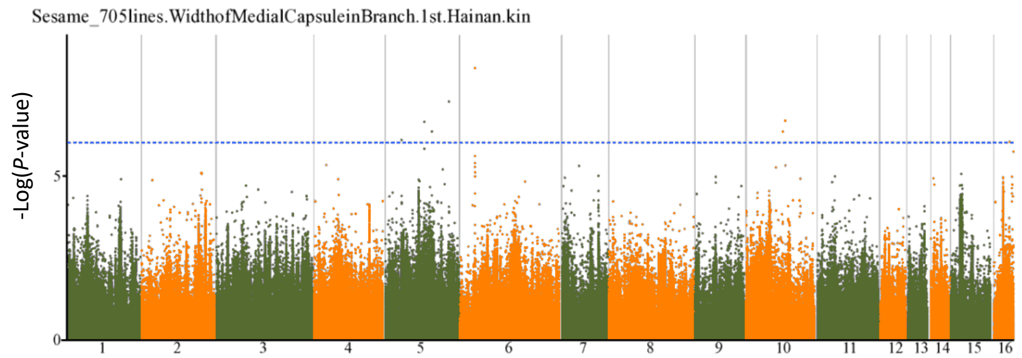

WMB-NN

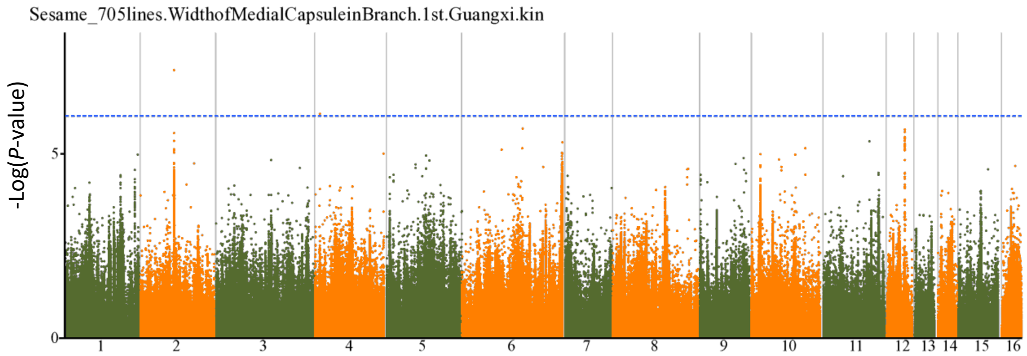

WMB-WH

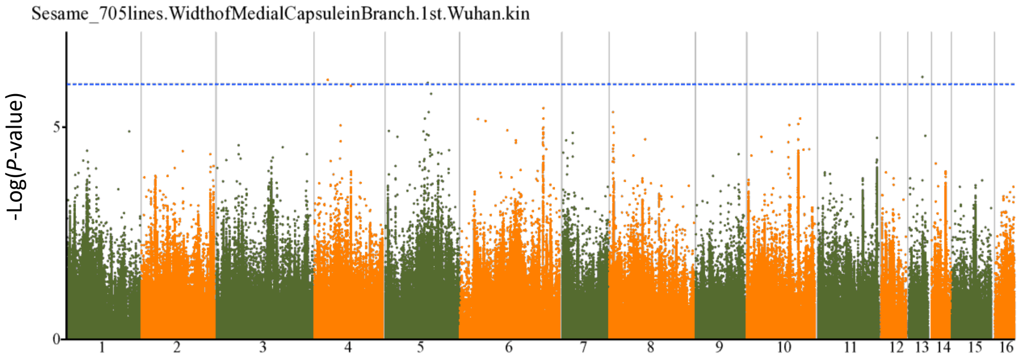

WMM-SY

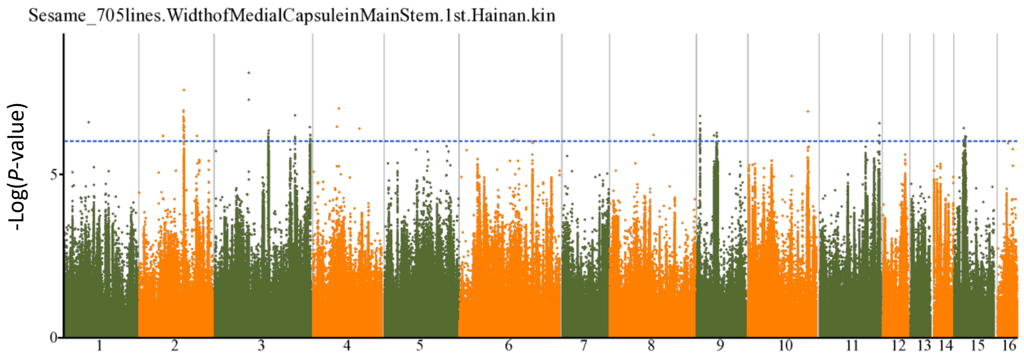

WMM-NN

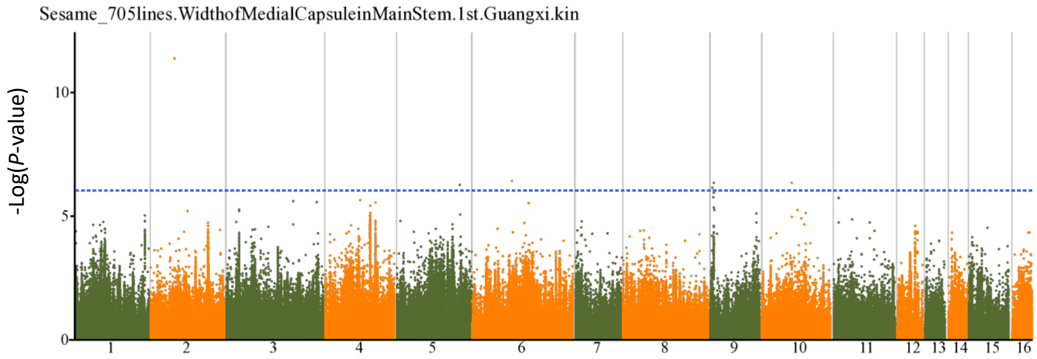

WMM-WH

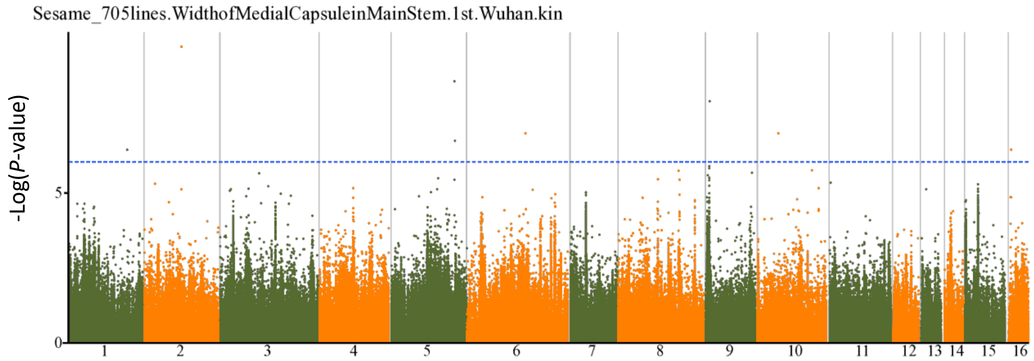

YB-SY

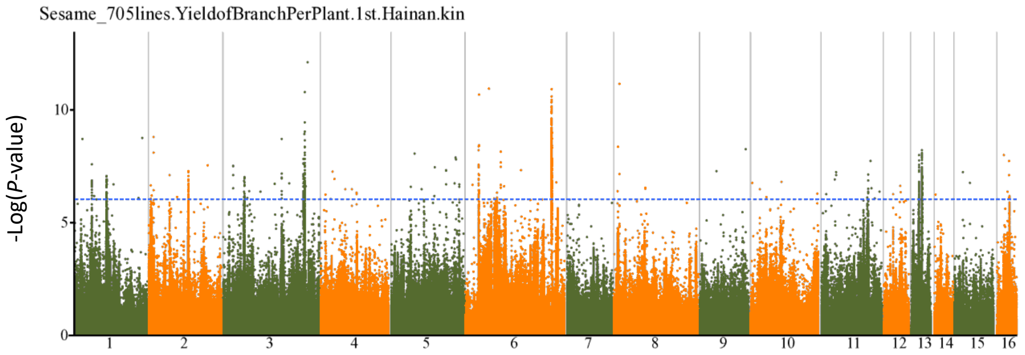

YB-NN

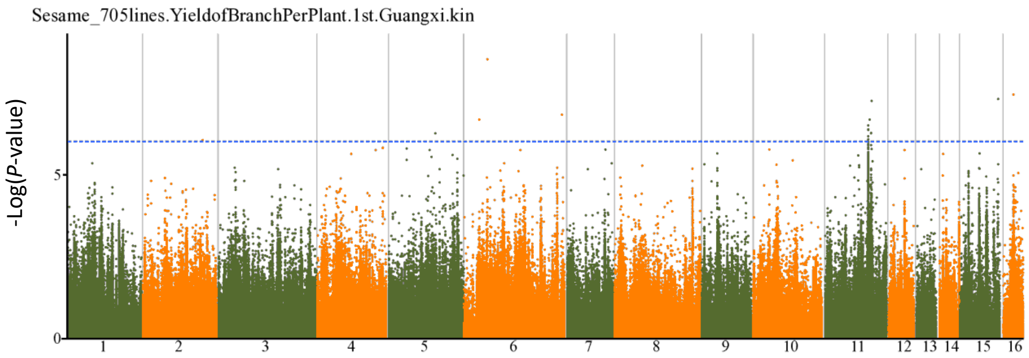

YB-WH

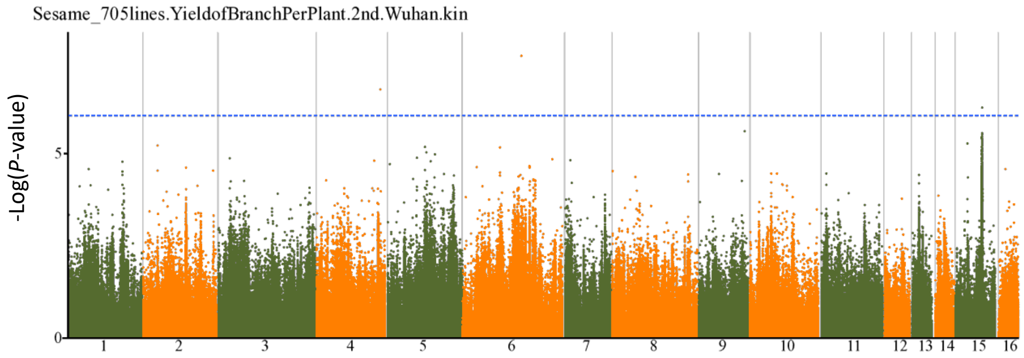

YLB-SY

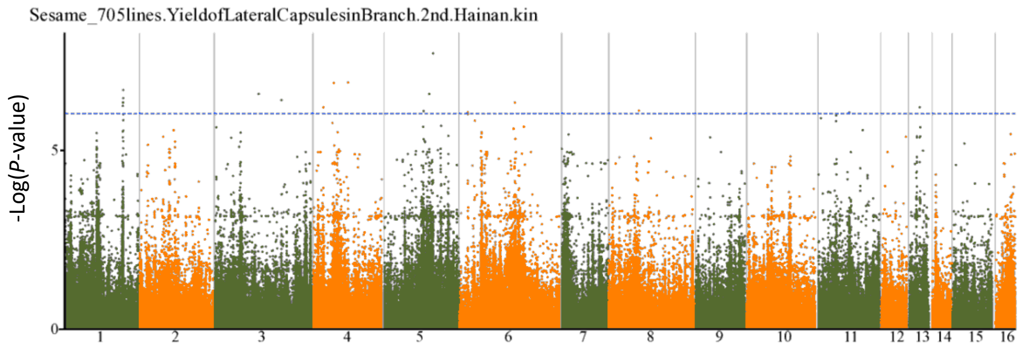

Sesame_705lines.YieldofLateralCapsulesinBranch.2nd.Hainan.kin
-Log(P-value)
0
5
1
2
3
4
5
6
7
8
9
10
11
12
13
14
15
16

YLB-NN

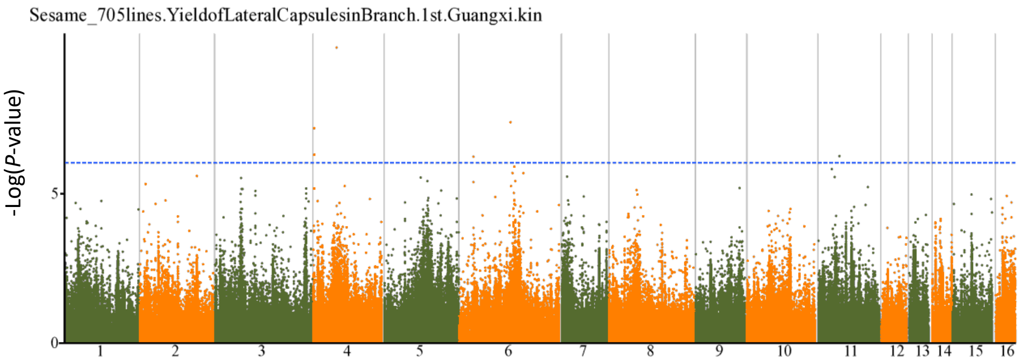

Sesame_705lines.YieldofLateralCapsulesinBranch.1st.Guangxi.kin
-Log(P-value)
0
5
1
2
3
4
5
6
7
8
9
10
11
12
13
14
15
16

YLM-SY

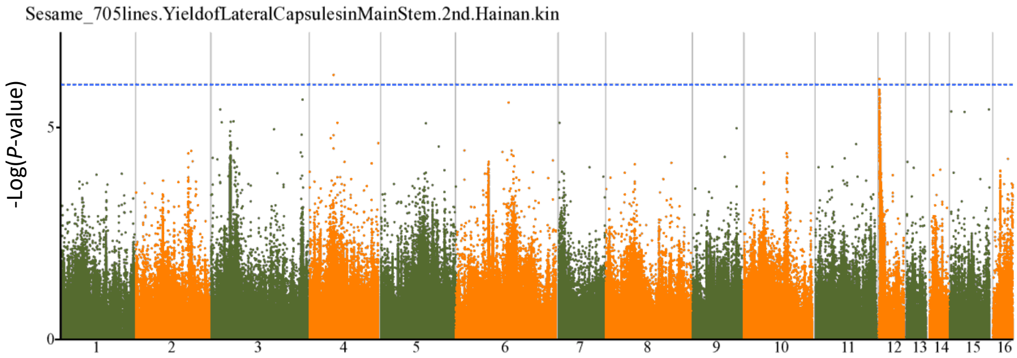

Sesame_705lines.YieldofLateralCapsulesinMainStem.2nd.Hainan.kin
-Log(P-value)
5
0
1
2
3
4
5
6
7
8
9
10
11
12
13
14
15
16

YLM-NN

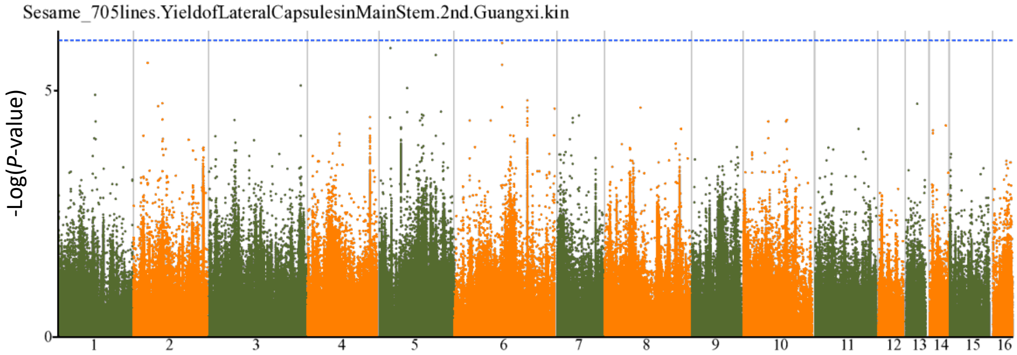

Sesame_705lines.YieldofLateralCapsulesinMainStem.2nd.Guangxi.kin
-Log(P-value)
5
0
1
2
3
4
5
6
7
8
9
10
11
12
13
14
15
16

YM-SY

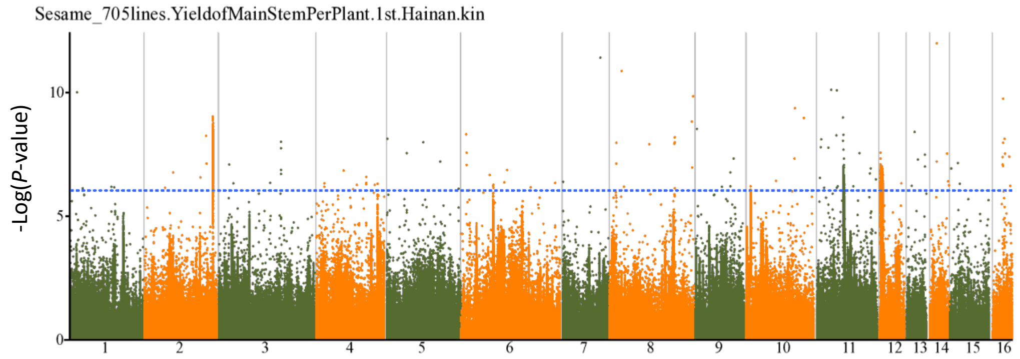

YM-NN

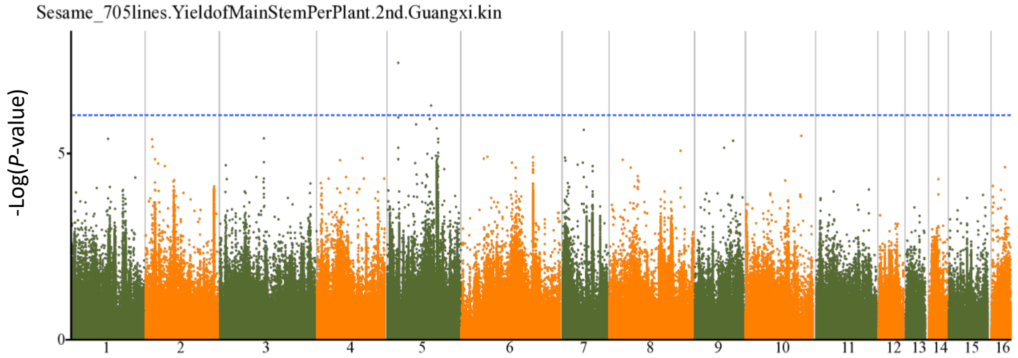

YM-WH

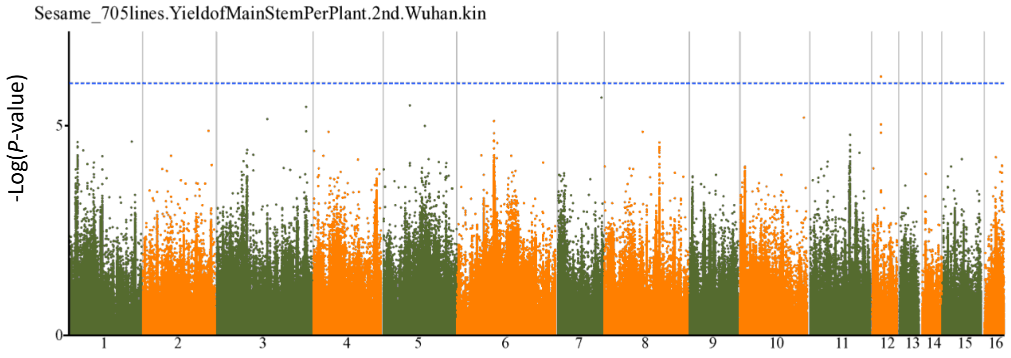

YMB-SY

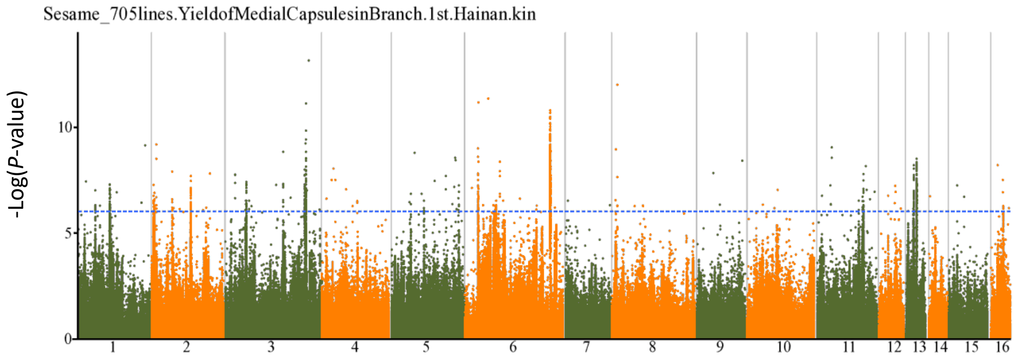
Sesame_705lines.YieldofMedialCapsulesinBranch.1st.Hainan.kin
-Log(P-value)
0
5
10
1
2
3
4
5
6
7
8
9
10
11
12
13
14
15
16

YMB-NN

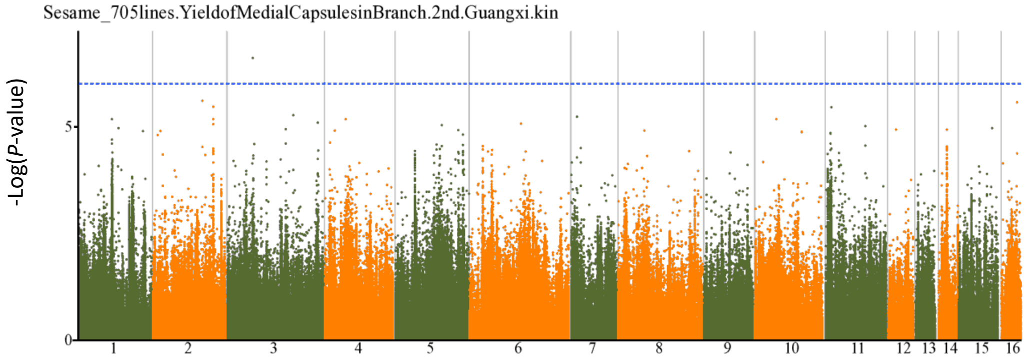
Sesame_705lines.YieldofMedialCapsulesinBranch.2nd.Guangxi.kin
-Log(P-value)
0
5
1
2
3
4
5
6
7
8
9
10
11
12
13
14
15
16

YMM-SY

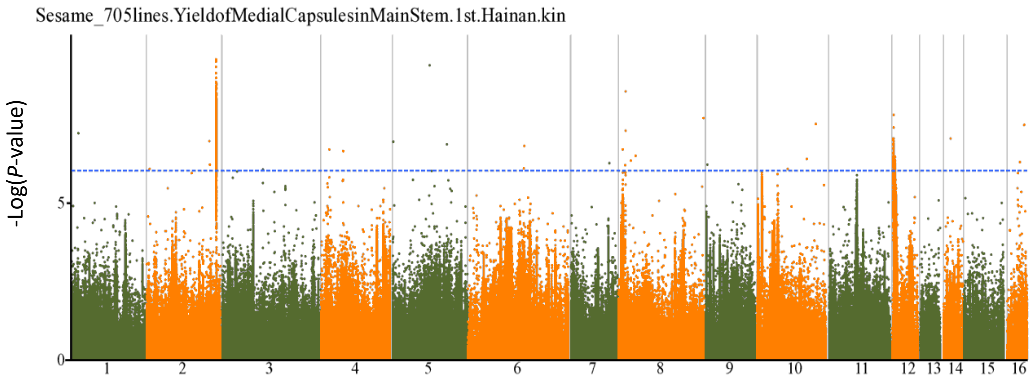
Sesame_705lines.YieldofMedialCapsulesinMainStem.1st.Hainan.kin
-Log(P-value)
0
5
1
2
3
4
5
6
7
8
9
10
11
12
13
14
15
16

YMM-NN

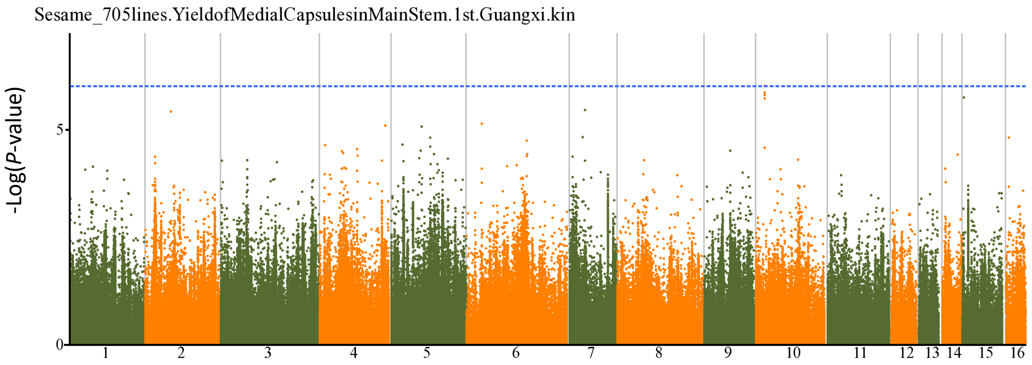
Sesame_705lines.YieldofMedialCapsulesinMainStem.1st.Guangxi.kin
-Log(P-value)
0
5
1
2
3
4
5
6
7
8
9
10
11
12
13
14
15
16

YP-SY

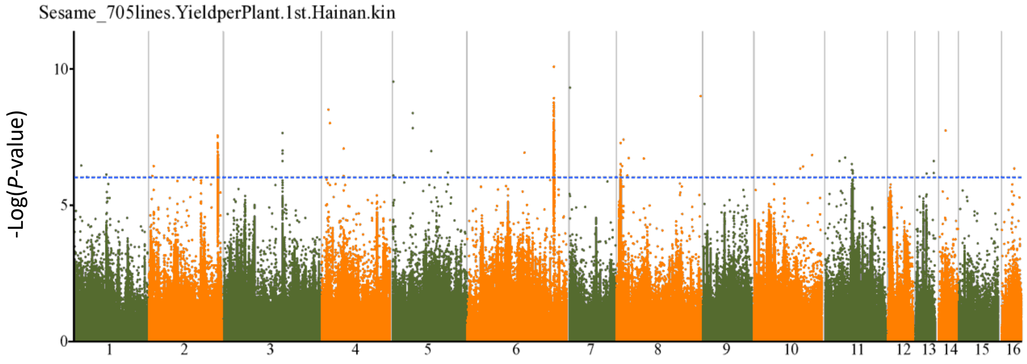

YP-NN

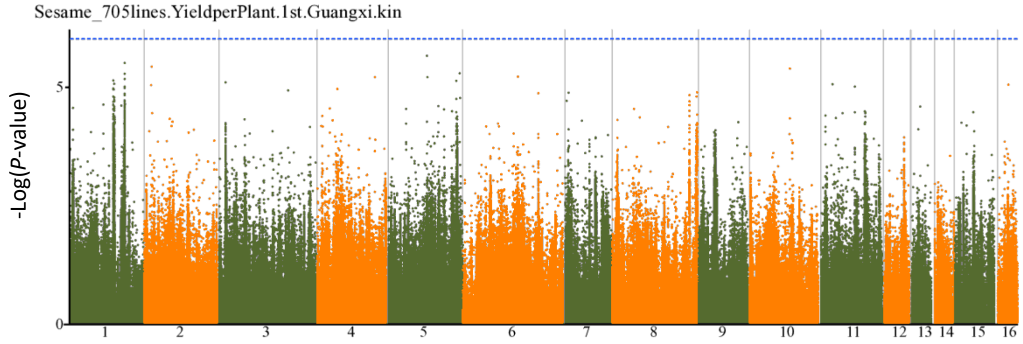

YP-WH

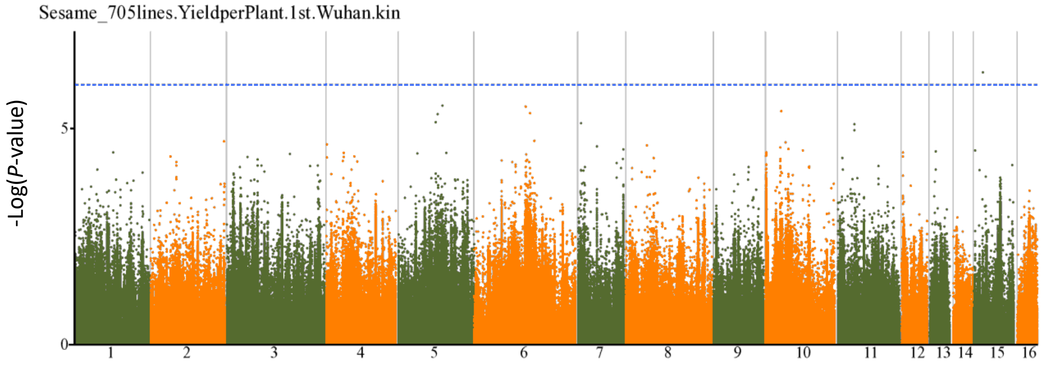

Supplement: Supplementary file 1 [file ijms-19-02794-s001.zip › Figure S2.pdf]
