# Supplementary figures and images for: Genome-Wide Association Studies of 39 Seed Yield-Related Traits in Sesame (Sesamum indicum L.)
Source: Int J Mol Sci. 2018 Sep 17;19(9):2794. doi: 10.3390/ijms19092794 (PMC6164633; doi:10.3390/ijms19092794)

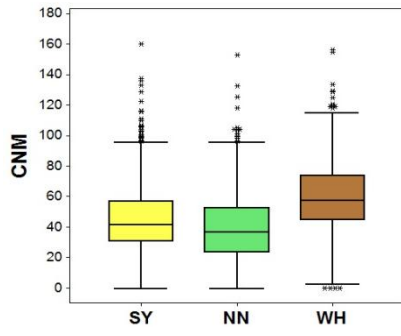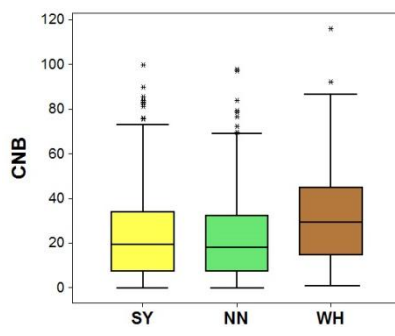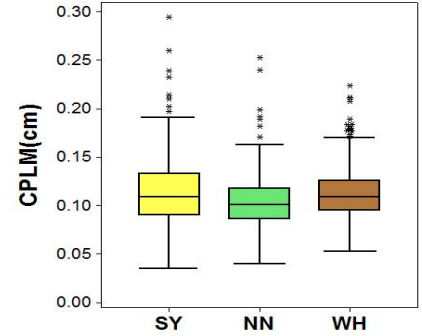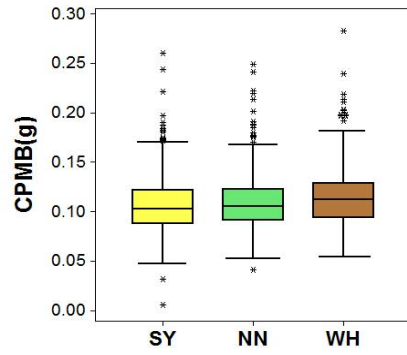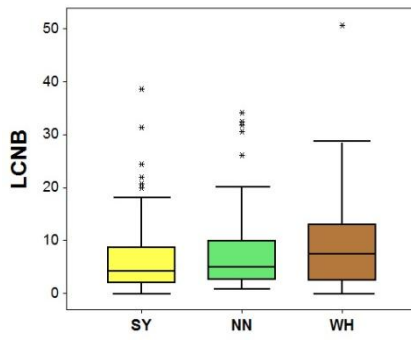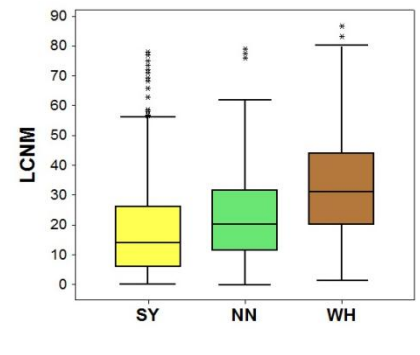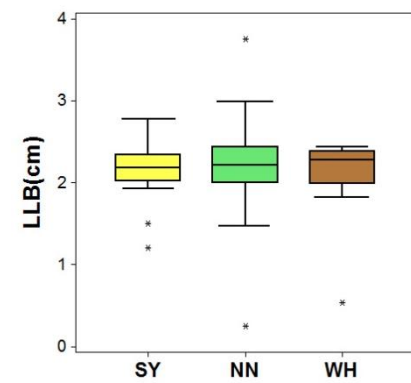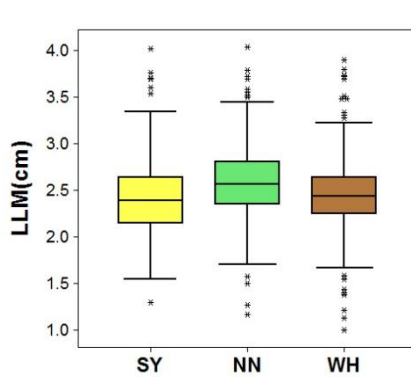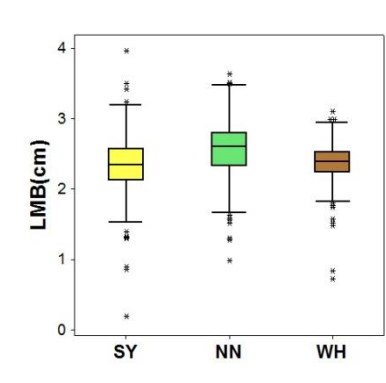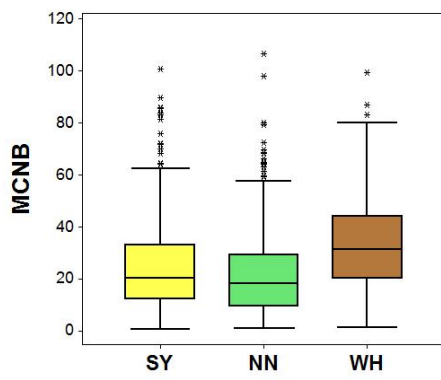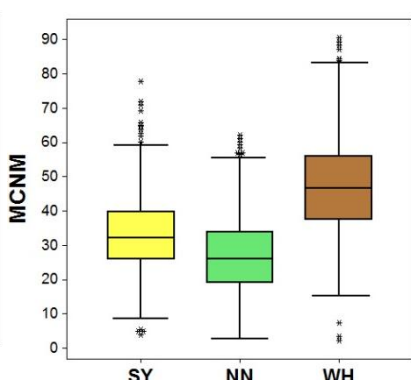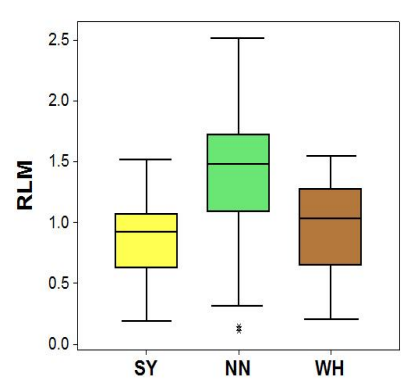

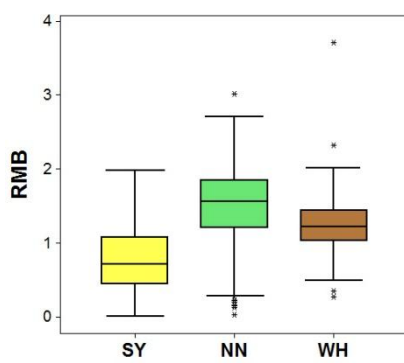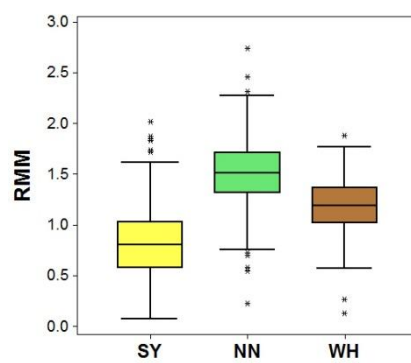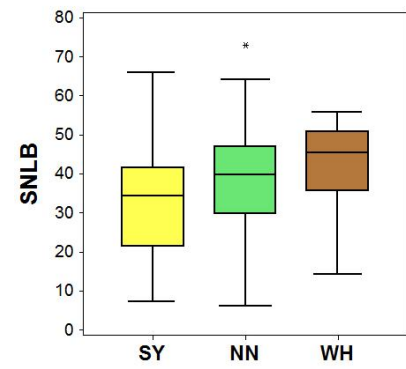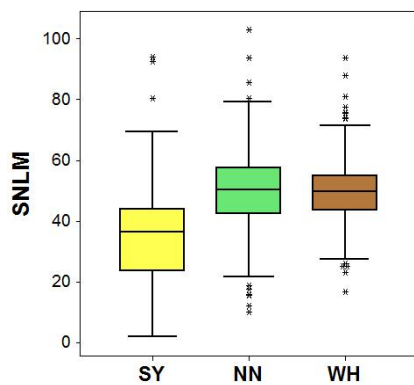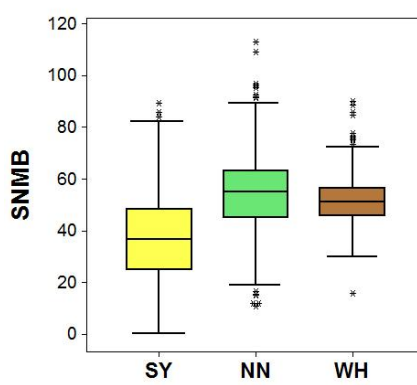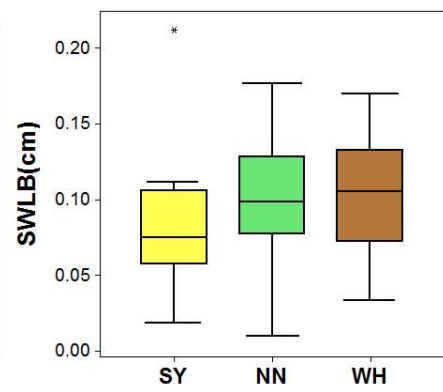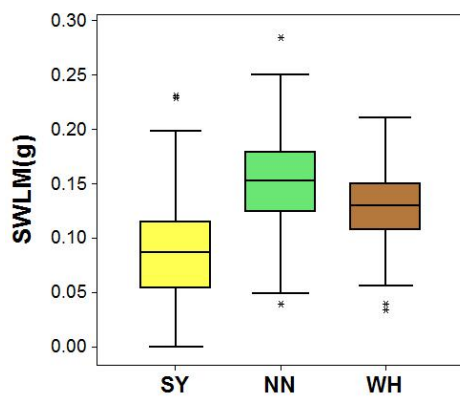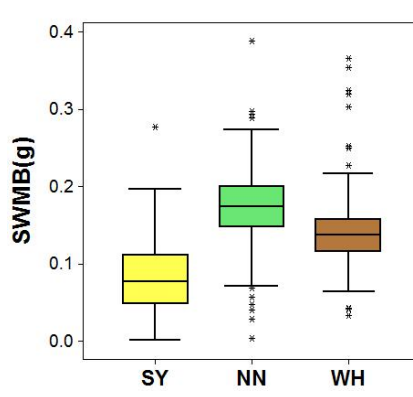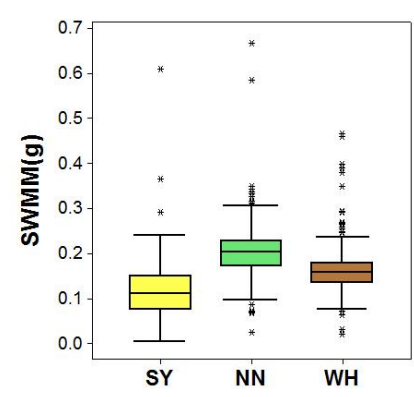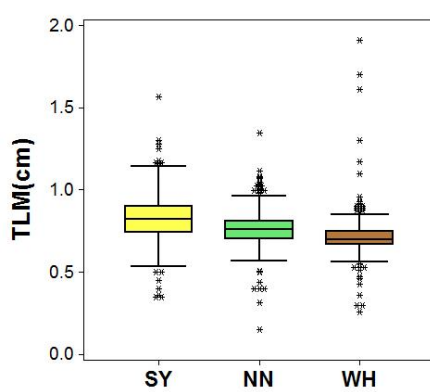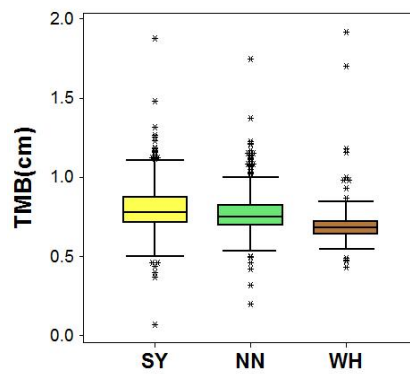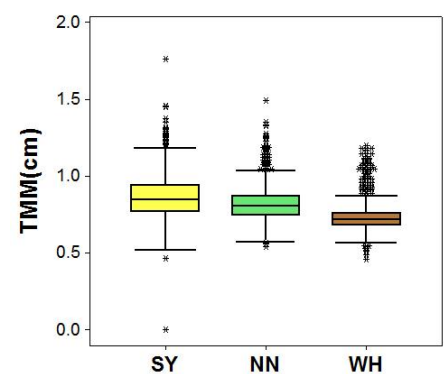

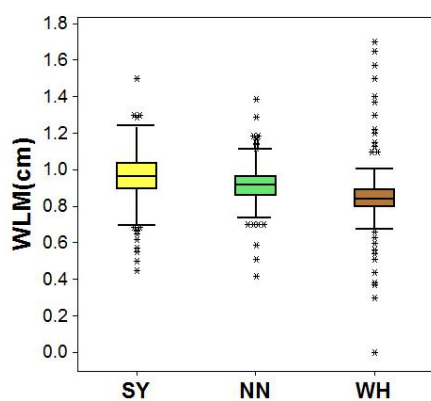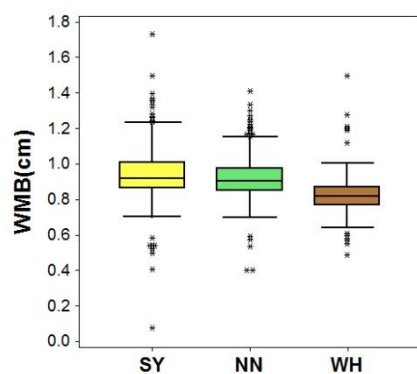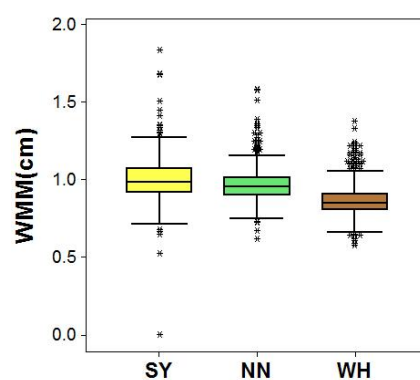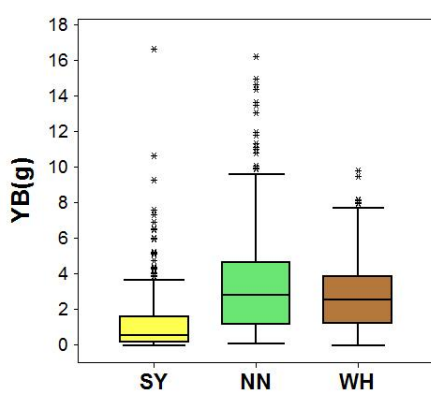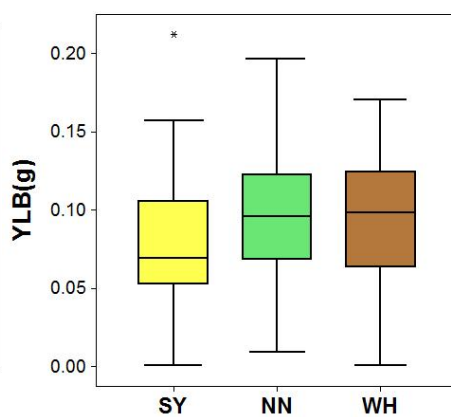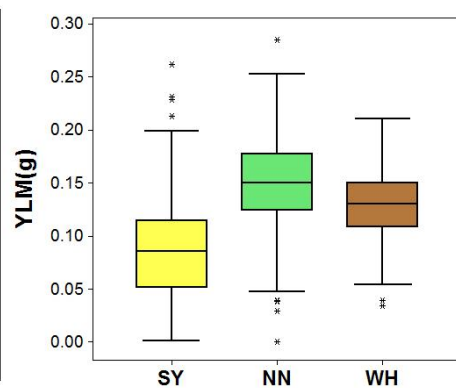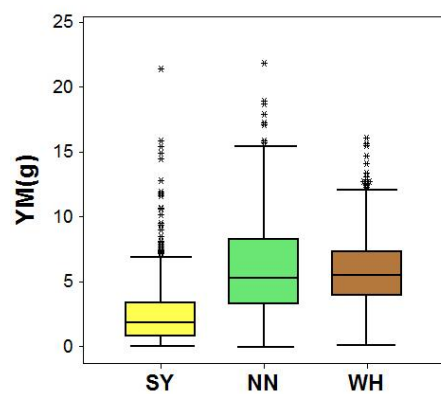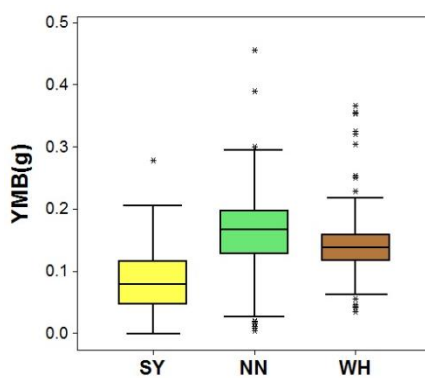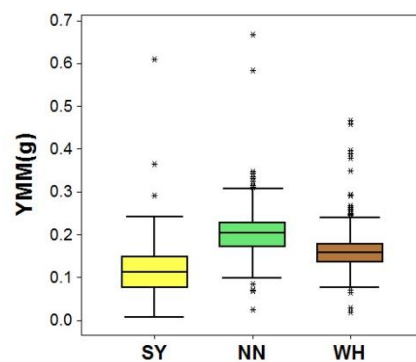

Supplement: Supplementary file 1 [file ijms-19-02794-s001.zip › Figure S1.pdf]
